# Activity Guided Azide-methyllysine Photo-trapping for Substrate Profiling of Lysine Demethylases

**DOI:** 10.1021/jacs.3c07299

**Published:** 2023-09-13

**Authors:** Jordan Kuwik, Kathryn Hinkelman, Megan Waldman, Kaitlyn E. Stepler, Shana Wagner, Simran Arora, Sasha Chernenkoff, Chino Cabalteja, Simone Sidoli, Renã AS Robinson, Kabirul Islam

**Affiliations:** † Department of Chemistry, University of Pittsburgh, Pittsburgh, Pennsylvania 15260, United States; ‡ Department of Chemistry, Vanderbilt University, Nashville, Tennessee 37235, United States; § Albert Einstein College of Medicine, Bronx, New York 10461, United States

## Abstract

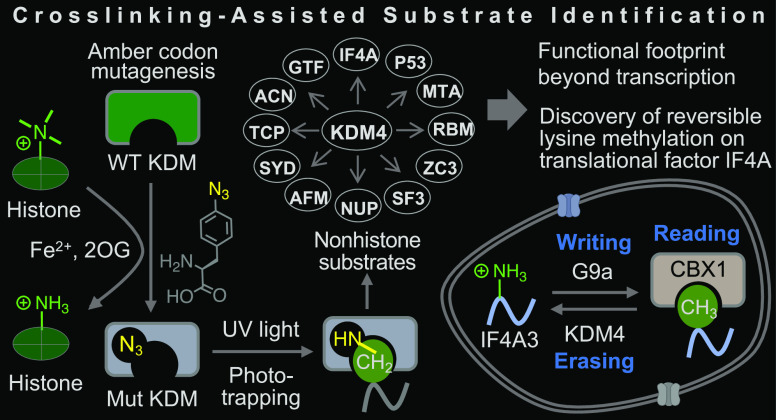

Reversible post-translational modifications (PTMs) are key to establishing protein–protein and protein–nucleic acid interactions that govern a majority of the signaling pathways in cells. Sequence-specific PTMs are catalyzed by transferases, and their removal is carried out by a class of reverse-acting enzymes termed “detransferases”. Currently available chemoproteomic approaches have been valuable in characterizing substrates of transferases. However, proteome-wide cataloging of the substrates of detransferases is challenging, mostly due to the loss of the epitope, rendering immunoprecipitation and activity-based methods ineffective. Herein, we develop a general chemoproteomic strategy called crosslinking-assisted substrate identification (CASI) for systematic characterization of cellular targets of detransferases and successfully apply it to lysine demethylases (KDMs) which catalyze the removal of methyl groups from lysine sidechain in histones to modulate gene transcription. By setting up a targeted azido-methylamino photo-reaction deep inside the active site of KDM4, engineered to carry *p*-azido phenylalanine, we reveal a novel “demethylome” that has escaped the traditional methods. The proteomic survey led to the identification of a battery of nonhistone substrates of KDM4, extending the biological footprint of KDM4 beyond its canonical functions in gene transcription. A notable finding of KDM4A-mediated demethylation of an evolutionarily conserved lysine residue in eukaryotic translational initiation factor argues for a much broader role of KDM4A in ribosomal processes. CASI, representing a substantive departure from earlier approaches by shifting focus from simple peptide-based probes to employing full-length photo-activatable demethylases, is poised to be applied to >400 human detransferases, many of which have remained poorly understood due to the lack of knowledge about their cellular targets.

## Introduction

**Figure 1:**
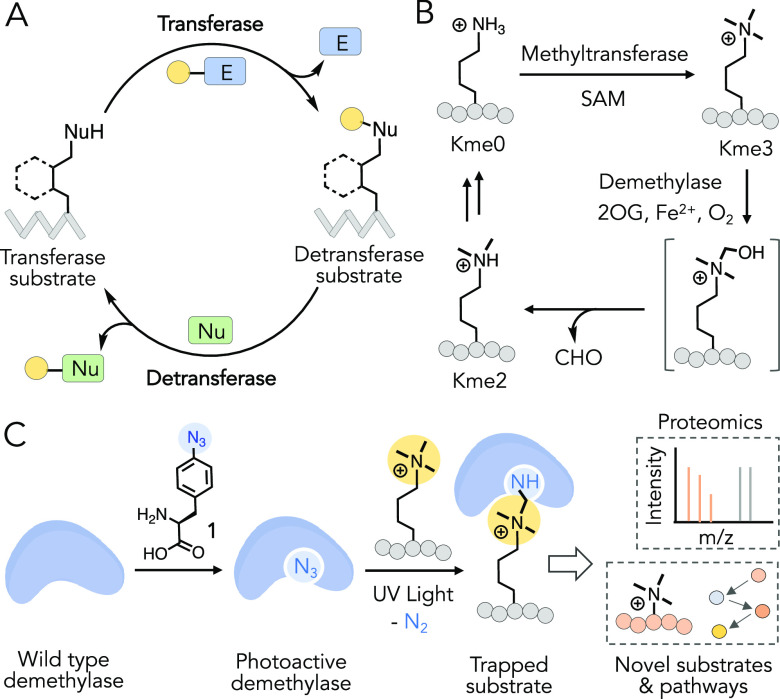
CASI for detransferases. (A) Transferase- and detransferase-mediated substrate modification controls most of the cellular processes. (B) Scheme showing lysine methylation and demethylation by KMT and KDM. (C) Schematic showing CASI steps. The exact site and nature of the crosslinking are undefined. SAM: S-adenosyl methionine, 2OG: 2-oxoglutarate, E: electrophile, Nu: nucleophile.

Reversible chemical modifications on DNA, RNA, and proteins establish a dynamic network of signaling pathways to regulate a majority of the cellular processes.^1^ Two types of biochemically opposing enzymes maintain the context-dependent status of such modifications: transferases (methyltransferase, acetyltransferase, kinase, ubiquitin ligase, etc.) and “detransferases” (demethylase, deacetylase, phosphatase, deubiquitinase, etc.) (Figure 1A). These enzymes act on diverse physiological substrates to carry post-synthetic information beyond what is encrypted in the primary sequence of biomolecules. Chemoproteomic approaches have made significant advances in cataloging substrates of the tranferases.^2,3^ In contrast, methods for identification of substrates of the “detransferases” are severely limited.^4,5^ Detransferase-mediated loss of epitope (a methyl/an acetyl/a phosphate group) renders the substrates inaccessible to affinity enrichment *via* bio-orthogonal ligation or immunoprecipitation. Unbiased profiling of substrates for >400 detransferases (200 phosphatases, 60 demethylases, 20 deacetylases, 100 deubiquitinases, etc.) encoded in the human genome has proved to be acutely challenging.^6−9^

A particularly important post-translational modification is the reversible histone methylation, which is catalyzed by lysine methyltransferases (KMTs) and lysine demethylases (KDMs) to regulate chromatin-dependent processes (Figure 1B).^10,11^ KDMs belong to the 2-oxoglutarate (2OG) and Fe^2+^-dependent dioxygenase superfamily, which catalyzes C–H oxidation on diverse substrates.^12^ Systematic proteomic analyses have identified the cellular “methylome”, consisting of >5000 human proteins with >10,000 unique methylation sites.^13^ In comparison to such well-established methylation events, KDM-mediated demethylation, particularly in nonhistone substrates, is poorly characterized (currently <50 identified on human proteins), despite their established role in signal transduction. Given that there are more than 40 KDMs present in the human proteome (Figure S1), the repertoire of cellular proteins undergoing lysine demethylation (the “demethylome”) is expected to be significantly larger.

Candidate-based studies have shown that KDM-mediated nonhistone demethylation can alter interacting partners, stability, and localization of proteins. Examples include KDM1-mediated demethylation of MYPT1 and Rb proteins to modulate E2F-responsive genes, critical for cell division cycle and oncogenesis;^14^ demethylation of pc2 by KDM4C for ncRNA-mediated reprograming of growth control genes constitutes a distinct mechanism for how KDM4C facilitates tumorigenesis independent of histone demethylation;^15^ KDM6B-mediated demethylation of ERα is critical for transcriptional activation of anti-apoptotic protein Bcl2 in breast cancer;^16^ and KDM7C-induced demethylation of transcription factor Runx2 promotes its binding to DNA in osteoblasts with a potential role in impaired bone development.^17^ Such analysis suggests that nonhistone demethylation could constitute a regulatory network similar to that of protein phosphorylation and acetylation, underscoring the importance of cataloging the substrates for individual KDMs.

*In vitro* approaches using purified proteins or peptides as a potential source of KDM substrates do not fully capture the wide-ranging low-abundant substrates of demethylases. Photo-crosslinkable variants of HDAC8, each carrying a *p*-benzoyl-l-phenylalanine (BzF) and trapping mutants of HDAC1, have been developed to identify novel substrates of deacetylases;^4,5^ representative substrates include HSP90 and TRIM28 of HDAC8 and CDK1 and MSH6 of HDCA1. The large size of the BzF moiety in the active site, however, may limit the recognition of diverse substrates. Herein, we introduce a chemoproteomic strategy called crosslinking-assisted substrate identification (CASI) which employs an engineered KDM carrying a photo-activatable amino acid in its active site for substrate capture and proteomic characterization (Figure 1C). With the KDM4 subfamily as a paradigm, we report that catalytically competent demethylase variants carrying *p*-azido phenylalanine (AzF) **1** form a UV-induced covalent bond with diverse methylated substrates present in human cells. Proteomic analysis of the affinity-purified, crosslinked proteins uncovered a panel of nonhistone proteins as novel substrates of KDM4. A subsequent mechanistic study with the newly identified noncanonical substrates revealed that the biological footprint of KDM4 indeed extends beyond its established role in transcription to non-chromatin processes such as nuclear transport and regulation of translational machinery. Our work introduces CASI as a general chemoproteomic platform for substrate profiling of detransferases and demonstrates its application to characterize the novel demethylome of KDM4 that has escaped the conventional approaches.

## Results and Discussion

### Engineering KDM4 Subfamily with Photo-crosslinkable Amino Acid

To demonstrate the feasibility of CASI, we focused on the catalytically active members of KDM4 subfamily. KDM4A-E are known to demethylate primarily trimethylated lysine 9 in histone H3 (H3K9me3) for gene activation.^18^ However, nonhistone demethylation by specific members of the KDM4 family is emerging as a key regulator of signaling pathways. For example, KDM4B, but not any other members of the family, demethylates AKT to oppose methylation-dependent AKT activation;^19^ demethylation of MyoD specifically by KDM4C activates the transcription factor by preventing methylation-dependent MyoD degradation.^20^ We engineered the KDM4 members with photo-responsive amino acid **1** for affinity purification and characterization of their nonhistone substrates from cellular milieu.

**Figure 2:**
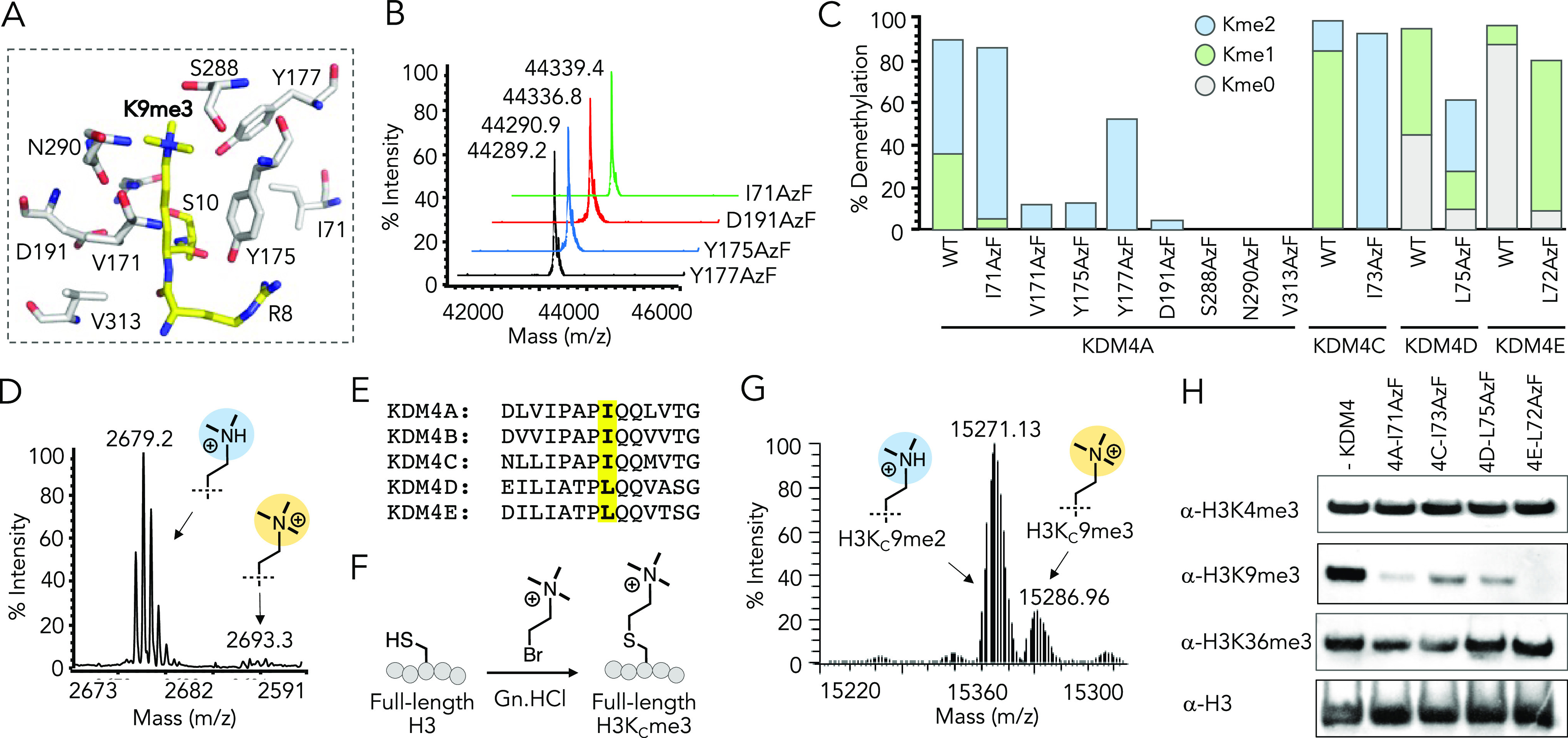
Engineering of KDM4 members for CASI. (A) Active site structure of KDM4A bound with K3K9me3 peptide (PDB: 2Q8C). Residues surrounding the trimethylated peptide have been subjected to replacement with AzF **1***via* amber suppressor mutagenesis. (B) ESI LC–MS spectra of selected KDM4A mutants carrying AzF. Spectra for the remaining mutants are provided in Figure S2. (C) Bar diagram showing % demethylation of H3K9me3 peptide **2** by wild-type KDM4A, C, D, and E and their respective AzF variants. The values are averages of two independent measurements by MALDI-TOF MS. A representative spectrum for demethylase activity of I71AzF mutant is shown in (D); the remaining MALDI-TOF spectra are displayed in Figure S3. (E) Sequence alignment of KDM4 subfamily shows I71 of KDM4A is conserved. (F) Synthesis of site-specifically introduced trimethylthialysine (K_C_me3) in full-length histone H3. (G) ESI LC–MS spectrum demonstrating demethylase activity of the I71AzF mutant of KDM4A on semi-synthetic H3K_C_9me3. (H) Distinct demethylase activity of KDM4A-I71AzF, KDM4C-I73AzF, KDM4D-L75AzF, and KDM4E-L72AzF mutants toward site-specifically methylated semi-synthetic histones, as observed by Western blot using modification-specific antibodies.

Structural studies of KDM4A-E have revealed that a series of conserved hydrophobic residues (I71, V171, Y175, Y177, and V313 for KDM4A) line up to recognize H3K9me3 at the active site (Figure 2A).^21^ Given that some of these residues are not engaged in direct interaction with either substrate (H3K9me3) or cofactor (2OG), we reasoned that these sites would be ideal for the introduction of AzF **1**. Furthermore, AzF being hydrophobic and structurally close to the amino acids, its incorporation into the active site is not expected to significantly alter substrate binding and catalysis. In addition to the advantage of minimal structural perturbation, AzF was selected because of efficient synthetic access, high crosslinking efficiency, and ease of incorporation into proteins in response to the amber suppressor codon (TAG).^22,23^ A few polar residues (D191, S288, and N290) were also selected for engineering because of their proximity to the trimethyllysine moiety in the active site. A panel of nine KDM4A mutants, site-specifically carrying **1,** were successfully expressed in *Escherichia coli* using evolved *M. jannaschii* TyrRS-tRNA_CUA_^Tyr^ pairs,^22^ purified to homogeneity, and confirmed by LC–MS (Figures 2B, S2, Table S1).

To identify KDM4A variants that are able to recognize and demethylate H3K9me3, we measured the loss of methyl groups using matrix-assisted laser desorption ionization time-of-flight mass spectrometry (MALDI-TOF MS).^24^ In our initial screening, the I71AzF mutant efficiently demethylated H3K9me3 peptide **2**, much like wild-type KDM4A; V171AzF, Y175AzF, and Y177AzF mutants were also found to be active, albeit to a lesser extent (Figures 2C,D, S3, Table S2). The lower activity likely stems from the changes in substrate binding because of the slight structural difference between AzF and the native amino acids. The I71AzF variant remained refractory toward H3K4me3 **3** and H3K27me3 **4** but demethylated the H3K36me3 peptide **5** modestly (Figure S4). These results are congruent with the reported enzymatic activity of wild-type KDM4A,^24−26^ thus confirming the retention of both site and sequence specificity in the engineered protein.

These results prompted us to develop the corresponding mutants for other KDM4 members. Amino acid sequence alignment of the active sites of KDM4A-E revealed that the residues targeted to engineer KDM4A are highly conserved across the subfamily (Figure 2E).^25,27^ We particularly focused on generating mutants equivalent to KDM4A-I71AzF. Using the evolved *M. jannaschii* TyrRS-tRNA_CUA_^Tyr^ pair, we successfully obtained KDM4C-I73AzF, KDM4D-L75AzF, and KDM4E-L72AzF mutant proteins and characterized them by liquid chromatography–mass spectrometry (LC–MS) (Figure S2, Table S1). In the MALDI-TOF-based demethylation assay, all three mutants showed catalytic activity to demethylate H3K9me3 peptide **2** akin to their KDM4A congener (Figures 2C, S3). Expression and purity of KDM4B-I72AzF were suboptimal for subsequent biochemical studies.

To examine if the active variants are able to demethylate full-length protein substrates, we introduced trimethylated thialysine (K_C_me3) at a specific position (K4, K9, or K36) in histone H3 *via* alkylation of each corresponding cysteine mutant with 2-bromoethyltrimethyl ammonium salt and confirmed the integrity of the semisynthetic histone by LC–MS analysis (Figures 2F, S5).^28^ Such site-specifically modified histone H3 is a known substrate of KDMs.^29^ Consistently, we observed that wild-type KDM4A demethylated full-length H3K9me3, which was separately prepared by treating H3 with methyltransferase Suv39H2 and SAM^30^ and semi-synthetic H3K_C_9me3 with equal efficiency (Figure S6). The I71AzF mutant efficiently demethylated H3K_C_9me3 to H3K_C_9me2 *in vitro* as revealed by electrospray ionization (ESI) LC–MS (Figure 2G). Furthermore, 4A-I71AzF, 4C-I73AzF, 4D-L75AzF, and 4E-L72AzF led to robust demethylation of H3K_C_9me3 but not H3K_C_4me3, as confirmed by Western blotting with a trimethyllysine-specific antibody (Figure 2H).

Importantly, H3K_C_36me3 was demethylated only by I71AzF and I73AzF, not by L75AzF and L72AzF, consistent with the fact that KDM4A and C, but not KDM4D and E, can demethylate H3K36me3, albeit to a lesser degree.^25,27^ These results demonstrate that we have successfully developed novel photo-activatable KDM4 variants employing amber suppressor mutagenesis and identified a mutant-carrying AzF at a conserved residue in the KDM4 subfamily with site- and sequence-specific histone demethylase activity akin to their wild-type congeners.

### Activity-Guided Photo-trapping of KDM4 Mutants and Histones

**Figure 3:**
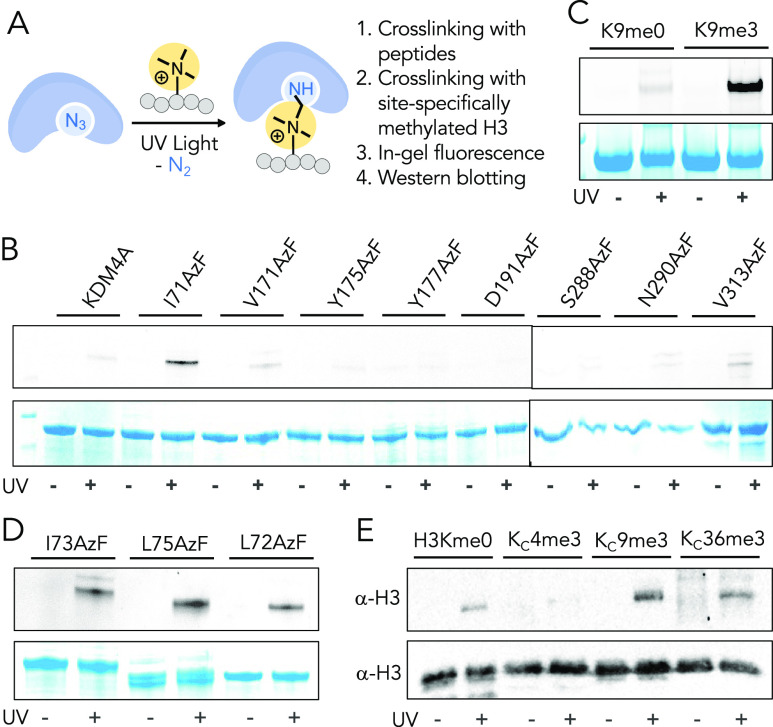
Crosslinking with histones. (A) Schematic showing crosslinking of engineered KDM4 to the methylated substrate (peptide or full-length histone H3), followed by fluorescence visualization for TAMRA-attached peptide and Western blotting for full-length histone. The exact site and nature of the crosslinking are undefined. (B) In-gel fluorescence (top panel) indicating crosslinking of wild-type KDM4A and its mutants to the TAMRA-H3K9me3 peptide **6**. Coomassie staining (bottom panel) is used as the loading control. (C) In-gel fluorescence (top panel) indicating the crosslinking of KDM4A-I71AzF to the TAMRA-H3K9me0 peptide **7** is negligible compared to the TAMRA-H3K9me3 peptide. (D) In-gel fluorescence (top panel) indicating the crosslinking of KDM4C-I73AzF, KDM4D-L75AzF, and KDM4E-L72AzF to the TAMRA-H3K9me3 peptide. (E) Immunoblotting using the H3 antibody, showing crosslinking of KDM4A-I71AzF to site-specifically methylated semi-synthetic, full-length histone H3. Bottom panel shows uncrosslinked H3 and serves as the loading control.

To further the development of CASI, which involves enzyme–substrate photo-trapping as a key step, we examined the KDM4 mutants for their ability to crosslink histone substrates (Figures 1C, 3A). The crosslinked enzyme–substrate pair is expected to be visualized by in-gel fluorescence as a single-component band. The tetramethylrhodamine (TAMRA)-attached H3K9me3 peptide **6** was photo-irradiated with individual KDM4A mutants using 365 nm light. The proteins were separated on polyacrylamide gel and visualized at 557 nm wavelength (λ_max_ for TAMRA) (Figures 3B, S7). Wild-type KDM4A lacking the crucial AzF moiety failed to undergo crosslinking despite having catalytic activity. Similarly, a majority of its catalytically dead AzF variants also proved to be ineffective to trap the substrate. The I71AzF mutant with efficient histone demethylase activity, on the other hand, underwent robust crosslinking with the histone peptide (Figure 3B). As expected, for control samples not exposed to UV light, no fluorescence band was detected on the gel, indicating the absence of crosslinking between mutants and the peptide. Furthermore, a TAMRA-labeled peptide **7** carrying H3K9me0, which is not a substrate of KDM4, showed negligible crosslinking with the mutant compared to H3K9me3, as visualized by in-gel fluorescence performed on the same gel (Figures 3C, S7). These results suggest that successful photo-trapping primarily relies upon binding of an authentic substrate into a catalytically poised active site, which is crucial for the activity-guided identification of substrates using CASI.

We also examined the crosslinking ability of KDM4C-I73AzF, KDM4D-L75AzF, and KDM4E-L72AzF toward the TAMRA-attached H3K9me3 peptide **6** (Figure 3D). As expected from their robust demethylase activity on **2**, all three mutants underwent successful crosslinking with the peptide upon UV irradiation, akin to KDM4A-I71AzF, demonstrating the generality of the activity-guided trapping of an enzyme–substrate complex among the KDM4 members.

To test the crosslinking efficiency with the full-length protein substrate, we irradiated KDM4A-I71AzF and the site-specifically methylated semisynthetic histones and immunoblotted them using the H3-specific antibody (Figure 3E). The mutant underwent crosslinking with H3K_C_9me3 and H3K_C_36me3, as judged by the appearance of a protein band of higher molecular weight only when the sample was subjected to photo-irradiation with 365 nm light. Importantly, H3K_C_4me3 failed to crosslink with the mutant, consistent with the observation that H3K4me3 is not a substrate of KDM4A. Together, the results demonstrate that the mutants with intact demethylase activity can trap the methylated histone peptide and site-specifically modified histones upon photo-irradiation without compromising sequence selectivity.

### Profiling Nonhistone Substrates of KDM4A

**Figure 4:**
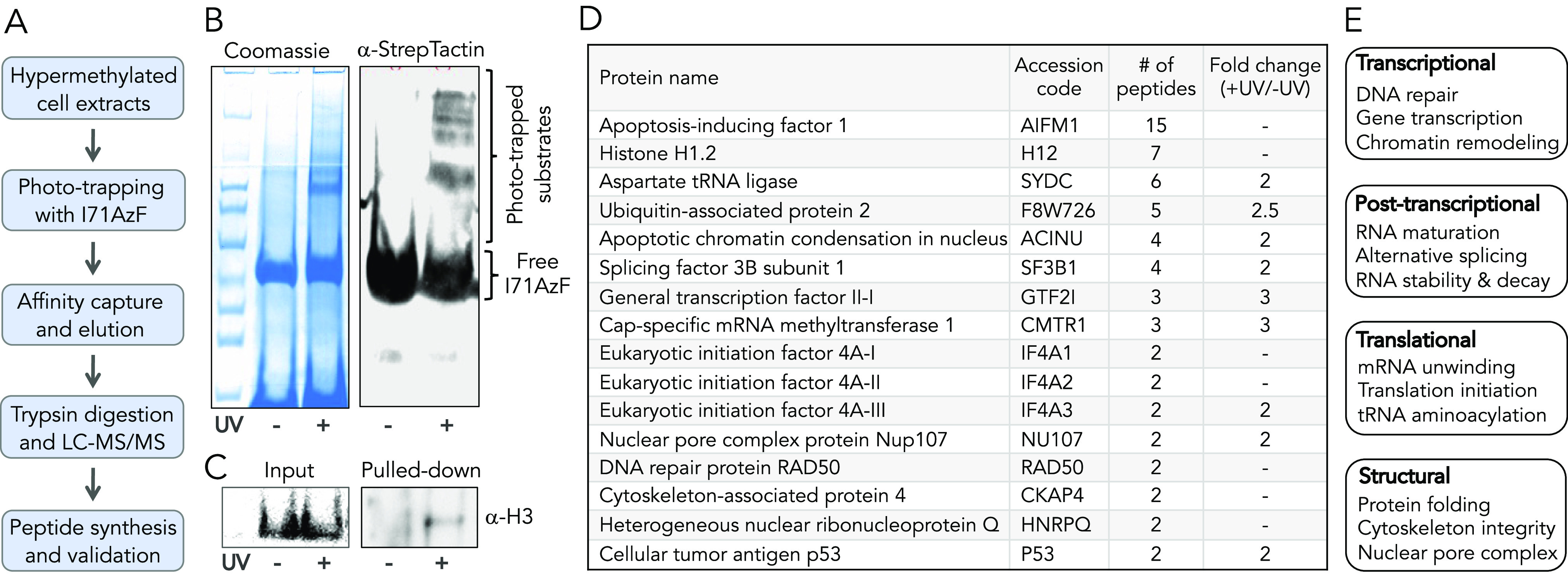
Proteomic characterization of KDM4A substrates. (A) Flow chart showing steps involved in crosslinking, enrichment, and characterization of KDM4A substrates present in HEK293T cells. (B) Coomassie staining of KDM4A substrates pulled down using StrepTactin beads from HEK293T cell extracts exposed to UV or kept in the dark. Western blot of the enriched samples using the anti-StrepTactin antibody. (C) Western blots of the input (prior to enrichment) and pulled-down (post-enrichment) samples using the anti H3 antibody show enrichment of H3 only in the UV-treated sample. (D) Representative KDM4A substrates revealed by CASI. Protein names, accession codes, # of peptides identified for each protein, and the fold-changes are given. No value in the fold change indicates the protein was present exclusively in the UV-treated sample; a complete list of putative substrates of KDM4A is provided in Table S3. (E) Biological functions of the putative substrates of KDM4A, suggesting KDM4A regulates a wide range of cellular processes *via* demethylation of the nonhistone substrates.

The ability of KDM4 mutants to bind and crosslink full-length histone suggests that the engineered proteins are suitable for capturing novel KDM4A substrates present in human cells. The known nonhistone substrates of KDM4 members and a recent observation that KDM4A is localized in the cytoplasm to associate with the translational machinery lend further support to the notion that the demethylases act on diverse cellular targets.^31,32^ For unbiased profiling of these substrates from cellular milieu, we cultured HEK293T cells in the presence of n-octyl-IOX1, a non-specific KDM4 inhibitor,^33^ generating a hypermethylated proteome. The whole-cell extract was incubated with Strep-tag containing the KDM4A-I71AzF protein and irradiated at 365 nm; the control sample was not exposed to UV light (Figure 4A). Crosslinked proteins were captured on StrepTactin beads, washed, and eluted with desthiobiotin. Polyacrylamide gel electrophoresis, followed by Coomassie staining and Western blotting of the eluted proteins using the anti-StrepTactin antibody revealed multiple bands of higher molecular weight in the UV-treated samples, suggesting successful crosslinking with putative substrates of KDM4A present in cell lysate (Figure 4B). To examine enrichment of a known substrate (*e.g.*, H3), we performed immunoblotting of the enriched samples with the H3 antibody. We observed that H3 was pulled down exclusively from the UV-irradiated sample although H3 was present in both UV-treated and non-treated samples prior to enrichment using StrepTactin beads. This result provides evidence for crosslinking of the I71AzF mutant with physiologically relevant known substrate of KDM4A such as H3 (Figure 4C).

We then carried out a systematic proteomic workflow to characterize the crosslinked species. The affinity-purified proteins present in each treatment group (±UV) were resolved on SDS-PAGE, extracted, and subjected to trypsin digestion. The tryptic peptides were analyzed by liquid chromatography–tandem mass spectrometry (LC–MS/MS). A total of 336 proteins were identified in UV-treated and non-treated samples (Table S3). We analyzed the data by applying two criteria in a sequential manner to find high-confidence substrates of KDM4A. First, exclusive unique peptides for each protein identified under a given condition (±UV) were averaged. Proteins which had a minimum of two unique peptides were selected. Applying this criterion, 43 proteins were eliminated from further analysis. We implemented a 2.0-fold enrichment filter based on average spectral counts obtained for each of the remaining 293 proteins. Proteins present in the UV-exposed sample that do not comply with the above criterion are not considered to be significantly enriched. Applying both criteria, we identified a total of 62 proteins as high-confidence nonhistone substrates of KDM4A, exclusively (24 proteins) or significantly (38 proteins) enriched by UV-mediated crosslinking (Figure 4D, Table S3). Prior to our study, candidate-based efforts could identify only a limited number (<10) of KDM4 substrates, which points toward the superiority of CASI for the unbiased profiling of proteome-wide substrates of the demethylase.

The presence of known substrates, such as histones H1 and H3, in the proteomic list consolidates the ability of CASI to identify authentic substrates of KDM4A.^34,35^ Analysis of the list of high-confidence proteins revealed that the candidate targets of KDM4A are of nuclear and cytosolic origin, which is consistent with the presence of KDM4 in both the nucleus and cytoplasm. The putative substrates are implicated in diverse cellular processes, including transcription, DNA repair, chromatin remodeling, nuclear export, splicing, nonsense-mediated decay, ribosome biogenesis, and protein synthesis, thus extending the functional footprint of KDM4A far beyond chromatin (Figure 4E). Particularly notable is nucleoporin 107 (NUP107), a component of nuclear pore complex, as a substrate of KDM4A because how reversible lysine methylation of NUP107 regulates nucleocytoplasmic transport is poorly understood. Lysine methylation is known to modulate transcriptional and pro-apoptotic activities of p53.^36,37^ KDM4-mediated demethylation on the tumor suppressor may constitute a novel switch for controlling its stability and promoter occupancy. Furthermore, consistent with an earlier report that KDM4A is associated with the translational machinery,^32^ we identified all the isoforms of eukaryotic translational initiation factor 4A (eIF4A) as KDM4A substrates, raising an intriguing possibility that KDM4A-mediated demethylation of eIF4A1-3 likely affects pre- and co-translational processes in a chromatin-independent manner. Together, the above results illustrate the steps involved in the development of the CASI proteomic platform and its application to identify non-canonical substrates of KDM4A that have remained refractory to traditional approaches.

### Biochemical Validation of Nonhistone Substrates of KDM4

We next examined if the KDM4 enzymes are capable of demethylating the high-confidence proteins identified using CASI. It has remained a major challenge to detect the actual crosslinked tryptic peptides owing to their low abundance, inefficient crosslinking, along with incomplete trypsin digestion of such non-natural substrates. Also, crosslinked species with an undefined chemical structure and composition pose a challenge to the existing proteomic tools for molecular analysis. To circumvent this issue, we surveyed the primary literature and publicly available proteomic databases (www.phosphosite.org; http://dbptm.mbc.nctu.edu.tw) and found that a majority of the putative substrates of KDM4 identified by CASI are either known or predicted to be methylated in eukaryotic cells.^36,38−43^ We reasoned that examining these Kme3 sites for KDM4-mediated demethylation *in vitro* would provide proof-of-concept validation of the CASI approach to identify *bona fide* substrates of the demethylases.

**Figure 5:**
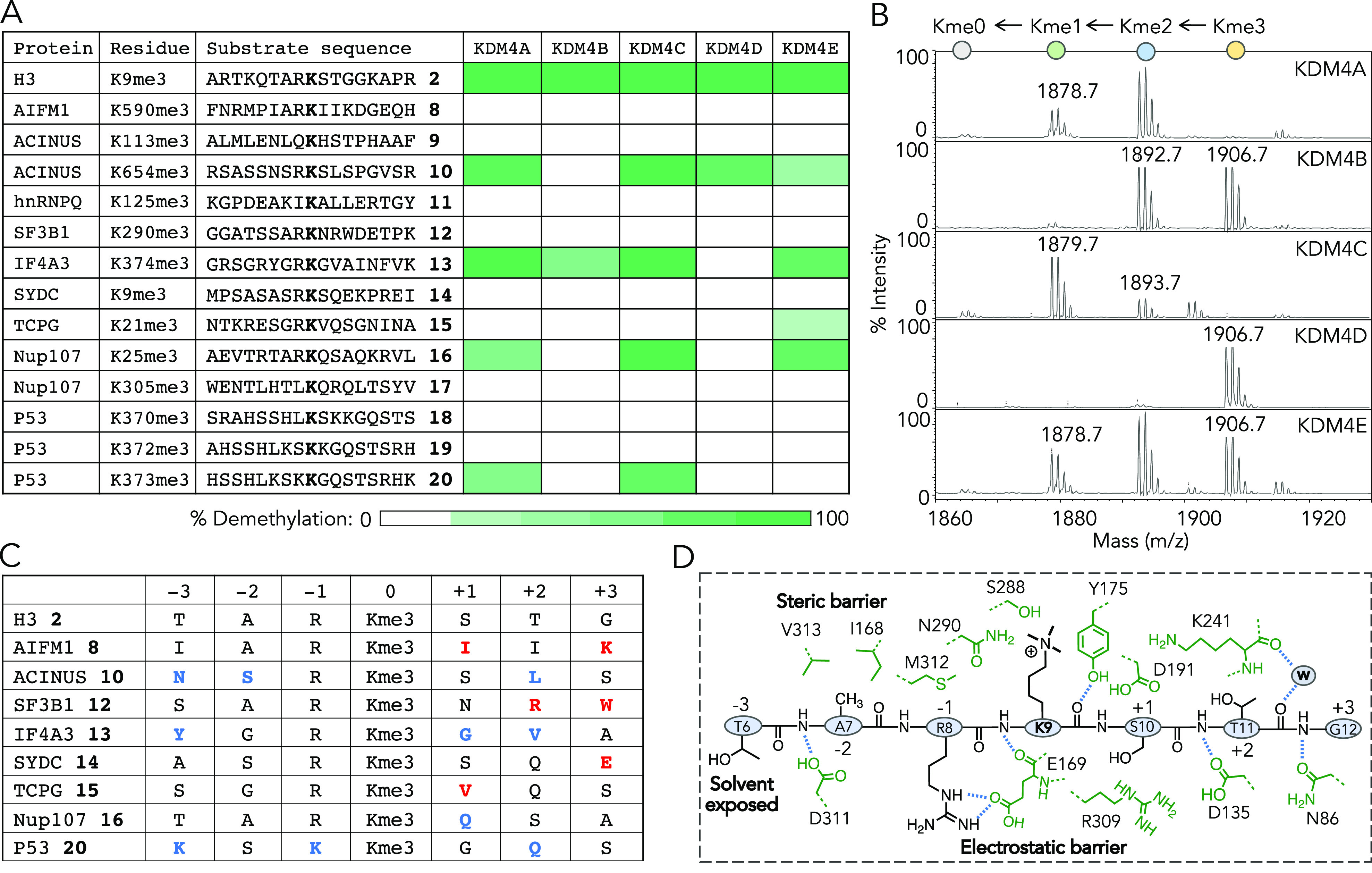
*In vitro* validation of KDM4 substrates. (A) Sequences of synthetic peptides **8–20** with a central trimethyllysine corresponding to a range of putative nonhistone substrates of KDM4A; H3K9me3 **2** is the positive control. Heat-map diagram showing % demethylation of peptides by wild-type KDM4A-E based on MALDI-TOF MS results. (B) Representative spectra showing demethylation of the eIF4A3-K374me3 **13** peptide by KDM4A-E; remaining MALDI-TOF spectra are displayed in Figure S8. (C) Amino acid preferences for KDM4A substrate selection. Colored (blue and red) amino acids are substantially different from those present in H3. Blue and red colors indicate allowed and restricted, respectively, at the specified position. A particular amino acid is colored only once for a given position across the peptides. (D) H3K9me3-bound KDM4A structure (PDB: 2Q8C) provides the rationale for substrate selection. Position −3 being solvent exposed provides a greater degree of flexibility; position −2 is housed in a tight hydrophobic pocket, allowing small amino acids (G/A/S) in the substrates; at −1 position, positively charged R or K is required due to strong electrostatic interaction with E169; smaller, uncharged amino acids are typically favored following the “RK” motif due to several charged residues (*e.g.*, D135, D191, K241, R309) in the active site; W: water.

We selected nine candidate proteins and synthesized a panel of thirteen peptides (**8–20**) using solid-phase chemistry, each carrying a central Kme3 residue guided by its known methylation site (Figure 5A, Table S2). To investigate the demethylase activity as well as specificity toward these nonhistone substrates, we included all the five catalytically active members of KDM4 subfamily. The enzymes demethylated the canonical substrate H3K9me3 **2** to a similar extent, confirming their biochemical integrity (Figures 5A, S3). However, they displayed varied degrees of lysine demethylation on the nonhistone peptides. Acinus-K654me3 **10**, eIF4A3-K374me3 **13**, Nup107-K25me3 **16**, and P53-K372me3 **20** peptides appeared to be promiscuous, as these peptides were demethylated by multiple KDM4s (Figures 5B, S8). In contrast, TCPG-K21me3 **15** was marginally demethylated only by KDM4E (Figures 5A, S7). Consistent with mono-and di-demethylase activity of KDM4s, each trimethylated substrate was demethylated once, leading to di- and mono-methylated peptides as the major products (Figures 5B, S8). Five out of nine proteins examined were identified as authentic substrates of KDM4, validating the proteomic findings. Systematic analysis with a larger set of proteins identified by CASI is expected to reveal additional substrates, distinct and overlapping, of the KDM4 subfamily.

We next analyzed the sequence preference of the demethylases toward nonhistone peptides. KDM4A-E members act primarily on the “RK” motif present in H3.^44^ The candidate substrates with this critical element (“RK”), including Acinus **10**, eIF4A3 **13**, and NUP107 **16**, underwent robust demethylation in our assay (Figure 5A,C). Consistently, peptides lacking an “RK” motif, such as Acinus **9**, hnRNPQ **11**, Nup107 **17**, and P53 **18** and **19**, failed to undergo demethylation by KDM4A-E. P53 **20** with a “KK” motif is also an active substrate, suggesting that an R or K at −1 position is required for the formation of strong electrostatic interactions with E169 in the active site of KDM4A (Figure 5C,D). We noted that a smaller amino acid (G/A/S) is preferred at position −2 that precedes “RK”, consistent with the “ARK” motif in H3 (Figure 5C). A tight hydrophobic pocket made of I168 and V313 provides a steric barrier to larger amino acids at −2 (Figure 5D). Position −3 is solvent exposed, allowing a higher degree of flexibility in size and charge, as reflected from the sequences of the active peptides **7** (NSRK), **10** (YGRK), and **17** (KSKK) (Figure 5C,D).

Despite carrying the required “A/GRK” motif, AIFM1 **8**, and SF3B1 **12** peptides, it failed to undergo demethylation, suggesting that amino acids following “RK” motif are also important for substrate selection. Smaller, uncharged amino acids are allowed at +1 as in the case for **10**, **13**, **16**, and **20**, while branched chains are restricted as observed for **8** and **15** (Figure 5A,C). We further noted that charged amino acids at positions +2 and +3 are unfavorable, likely due to the electrostatic barrier posed by D135, D191, and R309 (Figure 5C,D). This is consistent with the H3 sequence which carries smaller and neutral residues STG following “RK”. It is important to note that certain methylated peptides, such as **8**, **11**, **12**, and **14**, in the panel were not recognized as KDM4 substrates although the corresponding proteins were enriched by CASI. We reason that a larger set of peptides with preferred sequence motifs for each protein is to be evaluated to uncover the precise demethylation sites in the candidate substrates.

### Writing, Reading, and Erasing of eIF4A3 Methylation

**Figure 6:**
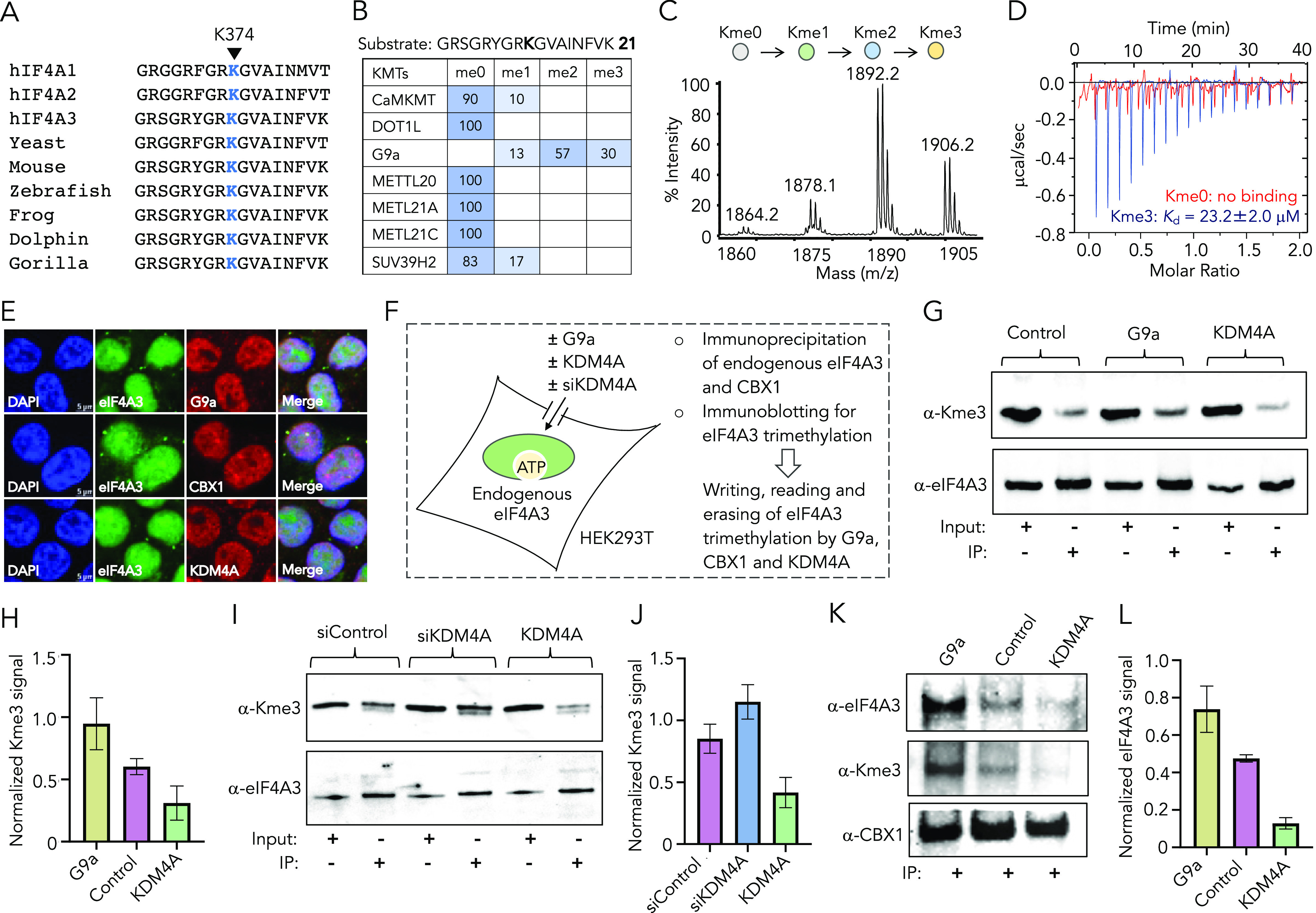
Writing, reading, and erasing of the trimethyllysine mark on eIF4A3. (A) Sequence alignment of human eIF4A1, 2, and 3 and from other species shows K374 of heIF4A3 is highly conserved among eukaryotes. (B) Heat-map diagram showing the extent of methylation of the eIF4A3-K374me0 peptide **21** by a range of lysine methyltransferases (KMTs) in the presence of S-adenosyl methionine (SAM) as judged by MALDI-TOF MS. (C) Representative MALDI-MS spectrum showing methylation of **21** by G9a. (D) ITC measurements of binding of CBX1 to eIF4A3-K374me0 **21** and eIF4A3-K374me3 **13** peptides. ITC isotherms for binding of CBX1 to eIF4A3-K374me1 **22** and eIF4A3-K374me2 **22** are given in Figure S10. Thermodynamic parameters are provided in Table S6. (E) Fixed cell immunofluorescence showing nuclear localization of eIF4A3, G9a, CBX1, and KDM4A. Biological replicates and quantification are provided in Figure S11. (F) Schematic showing the expression of full-length G9a and KDM4A, each in HEK293T cells, followed by immunoprecipitation (IP) of endogenous eIF4A3 and CBX1, followed by immunoblotting to examine writing, reading, and erasing of trimethylation mark on eIF4A3. (G) Western blot documenting successful writing and erasing of eIF4A3 methylation in mammalian cells. G9a led to increased Kme3 compared to the control vector (writing); KDM4A significantly reduced the Kme3 level compared to the control vector and G9a (erasing). Biological replicates are provided in Figure S12. (H) Bar diagram represents changes in eIF4A3 trimethylation based on data provided in Figures 6G and S12. (I) Western blot documenting knockdown of KDM4A using siRNA increases in eIF4A3 trimethylation compared to control siRNA; KDM4A overexpression robustly deceases eIF4A3 methylation. Biological replicates are provided in Figure S13. (J) Bar diagram showing quantitative changes in eIF4A3 trimethylation based on data provided in Figures 6I and S13. (K) Western blot documenting eIF4A3 enrichment by endogenous CBX1 (reading) as a function of trimethylation level. Enrichment of eIF4A3 increased in G9a-transfected cells and decreased in KDM4A-transfected cells compared to control cells. Biological replicates are provided in Figure S14. (L) Bar diagram showing quantitative changes in eIF4A3 enrichment based on data provided in Figures 6K and S14.

Members of the eukaryotic initiation factor 4A (eIF4A) belong to ATP-dependent RNA helicases of the DEAD-box family.^45^ eIF4A1 and 2 are cytoplasmic proteins that regulate the docking of mRNA on 40S ribosomal subunit during pre-initiation complex formation. eIF4A3 is nuclear and required for nonsense-mediated mRNA decay, a quality control system that degrades mRNAs containing premature termination codons.^46^ Using CASI, we identified all three members (eIF4A1-3) as putative substrates of KDM4A and subsequently validated that eIF4A3-K374me3 is indeed demethylated by KDM4A *in vitro*. Interestingly, K374 and its surrounding residues are conserved in eIF4A1 and 2, thus explaining their enrichment by the KDM4A-I71AzF mutant (Figure 6A). This result suggests that KDM4A likely demethylates eIF4A1-2 as well to regulate downstream translational processes.

To gain mechanistic insight into how reversible lysine methylation on the initiation factors is established, we sought to characterize the writer–reader–eraser axis involved in the pathway. Earlier studies showing eIF4A3 undergoes methylation by G9a *in vitro* and interacts with Chromobox protein 1 (CBX1),^38,43^ along with our observation of KDM4-mediated eIF4A3 demethylation, and point toward a dynamic “writing” (marking), “reading” (recognizing) and “erasing” (removing) of eIF4A3 methylation, much like the histone modifications. To identify the signaling axis, we synthesized the eIF4A3-K374me0 peptide **21** and subjected it to methylation by a representative set of KMTs, including CaMKMT, DOT1L, G9a, METTL20, METTL21A, METTL21C, and SUV39H2 (Figure 6B, Table S2). These KMTs, each with a distinct active site fold and catalytic mechanism, are known to methylate diverse histone as well as nonhistone proteins.^47,48^ Interestingly, G9a and SUV39H2 which act on H3K9me0 also methylated eIF4A3-K374me0 because of the sequence similarity between these two substrates (Figure 6B,C). The remaining KMTs, not reported as H3K9 methyl transferases, failed to methylate the eIF4A3-K374me0 peptide. While SUV39H2 monomethylated the eIF4A3 peptide, G9a led to di- and trimethylation (Figure 6C), an opposite trend of their activity observed for H3K9, suggesting G9a to be a writer of the eIF4A3-K374me3 mark. We also synthesized eIF4A3-K374me1 **22** and eIF4A3-K374me2 **23** peptides and observed that both are trimethylated by G9a (Figure S9, Table S2). Interestingly, **23** was demethylated to Kme1 by KDM4A; but **22** failed to convert to Kme0 (Figure S9), implying that exhaustive demethylation of eIF4A3-K374me3 likely requires additional demethylases, frequently observed for histone demethylation.

We next sought to identify a reader protein of eIF4A3-K374me3. Chromodomains are protein–protein interaction modules known to recognize the trimethylated lysine residue *via* a dedicated “aromatic cage”.^30,49^ Given that CBX1 binds H3K9me3 both *in vitro* and *in cellulo*, we determined the dissociation constant (*K*_d_) of CBX1 chromodomain from eIF4A3-K374me0 **21** and K394me3 **13** peptides using isothermal titration calorimetry (ITC) (Figures 6D, S10). To examine how the methylation state modulates binding, we also included eIF4A3-K374me1 **22** and eIF4A3-K374me2 **23** peptides. CBX1 chromodomain indeed recognized the trimethyllysine mark on eIF4A3 with a *K*_d_ of 23.2 ± 2.0 μM, much like H3K9me3 (Figure 6D). The extent of binding decreased for lower states of methylation with a *K*_d_ of 95.2 ± 1.4 μM for eIF4A3-K374me2 and no detectable interaction toward Kme0 and Kme1 peptides (Figures 6D, S10). Such results convincingly show that CBX1 can bind the nonhistone protein in a lysine trimethylation-specific manner.

To examine the “writing”, “reading,” and “erasing” of eIF4A3 trimethylation in a dynamic cellular environment, we first analyzed the localization of relevant proteins. Fixed-cell immunofluorescence imaging with appropriate antibodies revealed that eIF4A3, G9a, CBX1, and KDM4A are in the nucleus, indicating potential co-localization and interaction in cells (Figures 6E, S11). To gain further insight into their coordinated activity, we performed immunoprecipitation of endogenous eIF4A3 from HEK293T cells and immunoblotted with relevant antibodies (Figure 6F). In control vector-transfected cells, eIF4A3 underwent basal level lysine trimethylation, as evident from a *pan* tri-methyllysine antibody (Figures 6G, S12). We next performed eIF4A3 enrichment from cells individually expressing full-length G9a and KDM4A. Exogenous expression of G9a indeed led to a considerable increase in Kme3 on eIF4A3 compared to the basal level in non-transfected cells (Figures 6G,H, S12); overexpression of KDM4A, on the other hand, significantly reduced the Kme3 level, corroborating well with our *in vitro* data. We did not observe any changes in eIF4A3 enrichment across all three treatment groups. These results suggest that eIF4A3 undergoes lysine methylation and demethylation in cells by G9a and KDM4A, respectively.

To further confirm that KDM4A demethylates eIF4A3 in cells, we knocked down endogenous KDM4A in cultured HEK293T cells using short-interfering RNA (Figure S13). Western blot analysis revealed a significant loss of KDM4A protein. Subsequent immunoprecipitation of endogenous eIF4A3 from HEK293T cells and immunoblotting with the *pan* Kme3 antibody revealed a higher level of trimethylated eIF4A3 compared to control siRNA-treated cells (Figures 6I,J, S13). The moderate increase in Kme3 by KDM4A silencing is likely due to its demethylation by the remaining members of KDM4 subfamily in cells, consistent with our *in vitro* results (Figure 6J). Overexpression of wild-type KDM4A consistently led to robust eIF4A3 demethylation in HEK293T cells (Figure 6H,J).

**Figure 7:**
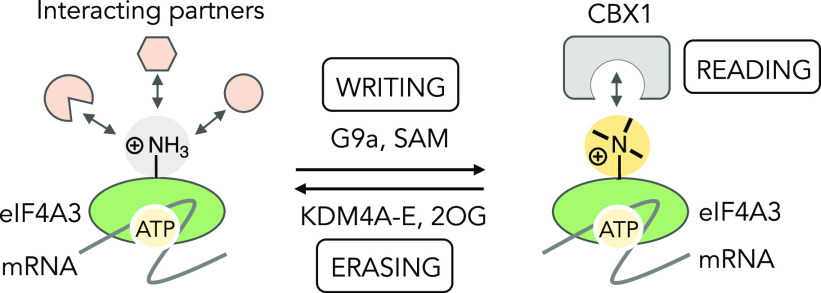
Illustration of writing, reading, and erasing of eIF4A3 methylation by G9a, CBX1, and KDM4, respectively, with potential implication in catalytic activity, ATP-dependent mRNA processing, protein–protein interaction, and localization of eIF4A3.

We next examined the ability of CBX1 to recognize trimethylated eIF4A3 in cells. Nuclear extracts from HEK293T cells expressing the control vector, G9a, and KDM4A were supplemented with recombinantly expressed CBX1 and subjected to affinity enrichment by using Ni-NTA-coated magnetic beads (CBX1 carries 6xHis in N-terminus), followed by immunoblotting with relevant antibodies. Western blot analysis clearly revealed trimethylation-dependent interaction between CBX1 and eIF4A3 (Figure S14). G9a-mediated eIF4A3 methylation led to a maximum enrichment of eIF4A3, while KDM4A decreased the association. Finally, eIF4A3 was immunoprecipitated from HEK293T cells using the CBX1 antibody. Western blot analysis confirmed that the two proteins (CBX1 and eIF4A3) indeed interact in cells, and the change in the eIF4A3 methylation level influenced its interaction with CBX1 (Figures 6K,L, S14). Overexpression of G9a led to robust enrichment of eIF4A3 by CBX1; in contrast, KDM4A reduced it compared to control vector-transfected cells (Figures 6K,L, S14). Collectively, these results provide strong evidence that this set of histone modifiers (G9a, CBX1, and KDM4A) can participate in dynamic “writing”, “reading”, and “erasing” of methyllysine on nonhistone proteins, such as eIF4A3, in cells. Intriguingly, eIF4A3-K374, which resides within the ATPase domain,^50^ is widely conserved among eukaryotes (Figure 6A), suggesting that reversible lysine methylation of the translational initiation factor constitutes a general mechanism to modulate its catalytic activity and localization for controlling post-transcriptional and ribosomal processes (Figure 7).

## Conclusions

Unbiased characterization of novel enzymes, substrates, and signaling pathways is critical for molecular understanding of biological processes. It has remained a significant technological challenge to profile substrates of the detransferase superfamily consisting of >400 members in humans. In this piece of work, we introduce a chemoproteomic approach called CASI that exploits UV-mediated trapping of substrates bound to engineered detransferases. A prominent feature of this strategy is the activity-guided crosslinking of authentic substrates deep inside the catalytic pocket of an enzyme. CASI represents a substantive departure from existing approaches which rely on either PMT-based immunoprecipitation, largely ineffective for detransferases due to the loss of the epitope, or crosslinking on the exposed protein surface, typically suffering from low efficiency due to quenching of the reactive species by the solvent molecules. Employing CASI, here we identify >60 potential nonhistone substrates of the KDM4A, a number significantly higher than what has been reported (<10) based on the candidate-based studies. The newly uncovered demethylome encompasses proteins of nuclear and cytosolic origin and suggests the regulatory function of KDM4 in nuclear export, mRNA decay, protein synthesis, and energy metabolism. We confirm several of the proteins, including eIF4A3, as authentic substrates of KDM4, demonstrating the robustness of CASI for substrate profiling. We discover a novel signaling axis involving a KMT (G9a), a chromodomain (CBX1), and a KDM (KDM4A) for writing, reading, and erasing of lysine methylation, respectively, on a translation factor (eIF4A3). How such reversible nonhistone methylation regulates catalytic and scaffolding activities of eIF4A3 and contributes to mRNA decay and ribosomal processes constitutes an important question to be investigated. Each newly revealed KDM4 substrate offers an avenue to explore the biological functions of reversible lysine methylation. We further anticipate that CASI, a structure-based protein engineering tactic, will find applications in other members of the detransferase superfamily for characterization of their substrates, signaling network, and downstream biological functions.

## Supporting Information Available

Methods for peptide synthesis and purification, expression and purification of proteins, biochemical assays, photo-crosslinking experiments, LC–MS and LC–MS/MS analyses, ITC experiments, results of the above experiments, phylogenetic analysis of human 2OG-dependent enzymes, calculated and experimental molecular weights of wild-type KDM4 proteins and their AzF mutants, list of synthesized peptides, list of genes used in the study, list of primers designed for site-directed mutagenesis, and thermodynamic parameters of CBX1 binding to eIF4A3 K374me0/1/2/3 peptides, as evaluated by ITC (PDF)
Analyzed proteomic data (XLSX)
