# Supplementary material for: Activity Guided Azide-methyllysine Photo-trapping for Substrate Profiling of Lysine Demethylases
Source: J Am Chem Soc. 2023 Sep 13;145(38):21066–76. doi: 10.1021/jacs.3c07299 (PMC10540216; doi:10.1021/jacs.3c07299)
Supplement: Supplementary file 1 — ja3c07299_si_001.pdf [file ja3c07299_si_001.pdf]

Activity Guided Azide-Methyllysine Phototrapping for Substrate Profiling of Lysine Demethylases

Jordan Kuwik,[1†] Kathryn Hinkelman,[1†] Megan Waldman,[1†] Kaitlyn E. Stepler,[2†] Shana Wagner,[1†] Simran Arora,[1] Sasha Chernenkoff,[1] Chino Cabalteja,[1] Simone Sidoli,[3] Renã AS Robinson,[2] and Kabirul Islam[1]*

[1]Department of Chemistry, University of Pittsburgh, Pittsburgh, PA USA 15260; [2]Department of Chemistry, Vanderbilt University, Nashville, TN USA 37235; [3]Albert Einstein College of Medicine, Bronx, NY, USA 10461.
[†]Equal contributions; *Corresponding author

Table of Content

1. General materials, methods, and equipment S2
2. Synthesis and purification of peptides S3
3. Expression and purification of wild type KDM4A-E S4
4. Mutagenesis, expression, and purification of AzF mutants of KDM4A-E S6
5. Expression and purification of histone H3 and its mutants S7
6. Chemical trimethylation of H3 mutants (semi-synthetic histones) S8
7. Expression and purification of lysine methyltransferases and chromodomains S8
8. Demethylase activity of wild type and AzF mutant proteins on peptide substrates S9
9. Demethylase activity of AzF mutants on semi-synthetic histones S10
10. Photo-crosslinking experiment with peptides and in-gel fluorescence S10
11. Photo-crosslinking experiment with semi-synthetic and extracted histones S10
12. Preparation of mammalian cell extracts for photo crosslinking S11
13. Photo-crosslinking with HEK293T cell extracts S11
14. LC-MS analysis of the intact H3 and KDM4 proteins S12
15. LC-MS/MS analysis of the affinity-purified proteins S12
16. Methylation activity of wild type KMTs on eIF4A3-K374me0/2/2 peptides S14
17. Isothermal Titration Calorimetry S14
18. Expression of full-length KDM4A in HEK293T cells S14
19. Knock down of KDM4A using siRNA S15
20. Fixed cell immunofluorescence imaging S16
21. Interactome pull-down using recombinant CBX1 protein S16
22. Immunoprecipitation using eIF4A3 and CBX1 antibodies S17
23. General protocol for Western blotting S18
24. Quantification of Western blots and nuclear proteins by Image J S20
25. Supplementary figures and tables S21
26. References S40

1. General materials, methods, and equipment

Chemicals: All chemicals were purchased from established vendors (e.g., Sigma-Aldrich, Acros Organics) and used without purification unless otherwise noted. Fmoc-Kme2-OH and Fmoc-Kme1(Boc)-OH were purchased from Chem-Impex International, Inc. (Fisher #NC1997908) and Sigma (#F4438-250MG), respectively. Optima grade acetonitrile was obtained from Fisher Scientific and degassed under vacuum prior to HPLC purification. All reactions to prepare 4-Azido phenylalanine (AzF) and Fmoc-Kme3-OH were carried out in round bottom flasks and stirred with Teflon®-coated magnetic stir bars under inert atmosphere when needed following reported methods.[1-3] Analytical thin layer chromatography (TLC) was performed using EMD 250 micron flexible aluminum backed, UV F_{254} pre-coated silica gel plates and visualized under UV light (254 nm) or by staining with phosphomolybdic acid, ninhydrin or anisaldehyde. Reaction solvents were removed by a Büchi rotary evaporator equipped with a dry ice-acetone condenser. Analytic and preparative HPLC was carried out on an Agilent 1220 Infinity HPLC with diode array detector. Concentration and lyophilization of aqueous samples were performed using Savant Sc210A SpeedVac Concentrator (Thermo), followed by Labconco or SP Scientific VirTis Benchtop Pro Freeze-Dryer system. Proton nuclear magnetic resonance spectra (^{1}H NMR) were recorded on Bruker Ultrashield™ Plus 600/500/400/300 MHz instruments at 24°C. Chemical shifts of ^{1}H and ^{13}C NMR spectra are reported as δ in units of parts per million (ppm) relative to tetramethylsilane (δ 0.0) or residual solvent signals: chloroform-d (δ 7.26, singlet), methanol-d_4 (δ 3.30, quintet), and deuterium oxide-d_2 (δ 4.80, singlet). Coupling constants are expressed in Hz. Mass spectra were collected on a Q-Exactive™ Thermo Scientific LC-MS with electron spray ionization (ESI) probe.

Plasmids, mutagenic primers, cell lines and antibodies: All the plasmids are for bacterial expression and obtained as gifts from individual laboratories or purchased from Addgene. Details of these constructs are given in Table S3. Mutagenic primers are obtained from Integrated DNA Technologies (Table S4). Various competent bacterial cells were used for protein expression and mutagenesis. Human embryonic kidney 293T (HEK293T) cells, obtained from the American Type Culture Collection (ATCC) and used in the current study following manufacturer's protocol. All the antibodies used in the current study are purchased from established vendors and used following manufacturer's protocol.

2. Synthesis and purification of peptides

H3K9me3 **2**, H3K4me3 **3**, H3K27me3 **4**, H3K27me3 **5**, TAMRA-H3K9me3 **6** and TAMRA-H3K9me0 **7** peptides were synthesized by the University of Pittsburgh Peptide Synthesis Facility. Crude peptides were purified using preparative reversed-phase HPLC (XBridge C18, 5 µm, 10 x 250 mm column), eluting with a flow rate of 4.00 mL/min and a gradient of acetonitrile starting from 0% v/v to 50% v/v in 12 min and then to 100% v/v by 18 min in aqueous trifluoroacetic acid (0.1% v/v). Purified peptides were concentrated by SpeedVac concentrator then lyophilized. Dried peptides were resuspended in water and stored at -20 °C before use. Concentrations of peptides with no aromatic amino acids were determined based on the observation that 1 mg/ml peptide generates an absorbance value (A_{205}) of 30 at 205 nm. Concentrations of peptides with aromatic amino acids were determined based on the absorbance and extinction coefficient for the aromatic amino acid at lambda max, using molar ratio of aromatic amino acid to peptide. The integrity of the purified peptides was confirmed by MALDI-TOF mass spectrometry.

Nonhistone peptides **8** - **23** were synthesized on solid support NovaPEG Rink Amide Resin (Sigma Aldrich #8550470005) in 5 mL fritted syringes. Amino acids were purchased from Sigma and used without further purification. Resin was swelled for a minimum of 20 minutes with N,N-Dimethylformamide (DMF) (Acros #348430025) prior to addition of C-terminal amino acid. Peptides were prepared at a 0.01-0.05 mmol scale. Four equivalents of amino acid, four equivalents of O-(1H-6-Chlorobenzotriazole-1-yl)-1,1,3,3-tetramethyluronium hexafluorophosphate (HCTU) (Sigma #8.51012), and six equivalents of N,N-Diisopropylethylamine (Sigma #496219) were first dissolved in 1-Methyl-2-pyrrolidinone (Sigma #443778) via sonication with an Ultrasonic Cleaner water bath (VWR #97043-968). The amino acid mixture was added to the resin and stirred under atmospheric conditions at 250rpm for 45-60 minutes. Resin was then washed with DMF three times prior to addition of 20% 4-methylpiperidine in DMF (Sigma #M73206). Deprotection solution was mixed with resin for 10 minutes at 250 rpm two times. Resin was then washed with DMF three times, and the next amino acid was added. Once the N-terminal amino acid was deprotected, the resin was washed three times each with DMF and dichloromethane (DCM) (Acros #348465000). The syringe and resin were dried in a HighVac desiccator for at least 30 minutes before proceeding. Cleavage cocktail containing 91.5% trifluoroacetic acid (TFA) (Alfa Aesar L063374), 2.5% thioanisole (Acros T28002), 2.5% phenol (Alfa Aesar #J64011), 2.5% deionized water, and 1% triisopropylsilane (Acros Organics # 214520100) was added to the resin. The sealed

fritted syringe was rocked for 2-4 hours at room temperature using a fixed angle rocker (Fisher 07202202). After cleavage, the solvent was ejected from the syringe into a 50mL Falcon tube, leaving behind the resin in the syringe. Nitrogen gas was bubbled through the solution to remove roughly 50% of the TFA prior to precipitation via -80°C diethyl ether for 5-10 minutes (Acros #326860010). Precipitate was then pelleted via centrifugation (Sorvall Legend XTR) at 3000 rpm for 5 mins. Supernatant was decanted. Crude peptide was then resuspended in 0.1% TFA prior to purification and characterization as described for peptides from the peptide facility. The integrity of the purified peptides was confirmed by MALDI-TOF MS. The complete list is in Table S1.

3. Expression and purification of wild type KDM4A-E

The N-terminal 6xHis-tagged human KDM4A-jmjC domain (catalytic domain of KDM4A) bacterial expression construct pNIC28-Bsa4 (Addgene ID: 38846) was obtained from Addgene and expressed following reported method.[4] The wild type KDM4A plasmid was transformed into *E. Coli* BL21 (DE3) competent cells (Invitrogen) using pNIC28-Bsa4 kanamycin-resistant vector. A single colony was picked up and grown overnight at 37°C in 10 mL of Luria-Bertani (LB) broth in the presence of 50 µg/mL kanamycin and 35 µg/mL chloramphenicol. The culture was diluted 100-fold and allowed to grow at 37°C to an optical density (OD_{600}) of 0.8, and protein expression was induced overnight at 17°C with 0.6 mM IPTG in an Innova 44® Incubator shaker (New Brunswick Scientific). Proteins were purified as follows: harvested cells were resuspended in 15 mL lysis buffer (50 mM Tris-HCl pH 8.0, 200 mM NaCl, 5 mM *β*-mercaptoethanol, 10% glycerol, 25 mM imidazole, Lysozyme, DNase, and Roche protease inhibitor cocktail). The cells were lysed by pulsed sonication (Qsonica-Q700), and centrifuged at 13000 rpm for 40 min at 4 °C. The soluble extracts were subject to Ni-NTA agarose resin (Thermo) according to manufacturer's instructions. After passing 20 volumes of washing buffer (50 mM Tris-HCl pH 8.0, 200 mM NaCl, 5 mM *β*-mercaptoethanol, 10% glycerol, and 25 mM imidazole), proteins were eluted with a buffer containing 50 mM Tris-HCl pH 8.0, 200 mM NaCl, 5 mM *β*-mercaptoethanol, 10% glycerol, and 400 mM imidazole. Proteins were further purified by size exclusion chromatography (Superdex-200) using AKTA pure FPLC system (GE healthcare) with buffer containing 50 mM Tris-HCl pH 8.0, 200 mM NaCl, and 10% glycerol. Purified proteins were concentrated using Amicon Ultra-10k centrifugal filter device (Merck Millipore Ltd.). The protein concentration was determined

using Bradford assay kit (BioRad Laboratories) with BSA as a standard. The concentrated proteins were stored at -80°C before use.

The N-terminal strep-tagged human KDM4B and KDM4D jmjC catalytic domain for bacterial expression were kindly provided by Prof. Raymond Trievel, University of Michigan.[5,6] The native constructs were transformed into *E. coli* Rosetta [DE3] competent cells. A single colony was picked up and grown overnight at 37°C in 10 mL of Luria-Bertani (LB) broth in the presence of 100 μg/mL ampicillin and 35 μg/mL chloramphenicol. The culture was diluted 100-fold and allowed to grow at 37°C to an optical density (OD_{600}) of 0.8, and protein expression was induced overnight at 18°C with 1 mM IPTG in an Innova 44® Incubator shaker (New Brunswick Scientific). Proteins were purified as follows: harvested cells were resuspended in 15 mL lysis buffer (50 mM Tris-HCl pH 8.0, 200 mM NaCl, 5 mM *β*-mercaptoethanol, 10% glycerol, Lysozyme, DNase, and Roche protease inhibitor cocktail). The cells were lysed by pulsed sonication (Qsonica-Q700) and centrifuged at 13000 rpm for 40 min at 4 °C. The soluble extracts were subject to Strep-tactin resin (Qiagen, cat# 30004) according to manufacturer's instructions. After passing 20 volumes of washing buffer (50 mM Tris-HCl pH 8.0, 200 mM NaCl, 5 mM *β*-mercaptoethanol, 10% glycerol), proteins were eluted with a buffer containing 50 mM Tris-HCl pH 8.0, 200 mM NaCl, 5 mM *β*-mercaptoethanol, 10% glycerol, and 5 mM D-desthiobiotin. Proteins were further purified by size exclusion chromatography (Superdex-200) using AKTA pure FPLC system (GE healthcare) with buffer containing 50 mM Tris-HCl pH 8.0, 200 mM NaCl, and 10% glycerol. Purified proteins were concentrated using Amicon Ultra-10k centrifugal filter device (Merck Millipore Ltd.). The protein concentration was determined using Bradford assay kit (BioRad Laboratories) with BSA as a standard. The concentrated proteins were stored at -80°C before use.

The N-terminal 6xHis-tagged human KDM4C catalytic domain construct for bacterial expression was a kind gift from Prof. Danica Fujimori, UCSF.[7] Plasmids were transformed into BL21 [DE3] star cells and grown at 37°C to an optical density of 0.6. Protein expression was induced with 0.3 mM IPTG overnight at 17°C. Following bacterial cell lysis, expressed protein was purified first by nickel affinity chromatography and secondly by size exclusion chromatography using a superdex

200 FPLC column. The proteins were concentrated via centrifugation. Protein concentration was determined using Bradford assay.

The N-terminal 6xHis-tagged KDM4E construct for bacterial expression was purchased from Addgene (#38990) and expressed following reported method.[8] The wild type KDM4E plasmid was transformed into *E. Coli* BL21 (DE3) competent cells (Invitrogen) using pNIC28-Bsa4 kanamycin-resistant vector. A single colony was grown overnight at 37°C in 10 mL of Luria-Bertani (LB) broth in the presence of 50 μg/mL kanamycin and 35 μg/mL chloramphenicol. The culture was diluted 100-fold and allowed to grow at 37°C to an optical density (OD_{600}) of 0.8, and protein expression was induced overnight at 17°C with 1 mM IPTG in an Innova 44® Incubator shaker (New Brunswick Scientific). KDM4E was purified from pelleted cells by resuspending in lysis buffer (50 mM Tris-HCl pH 8.0, 200 mM NaCl, 5 mM β-ME, 10% glycerol, 25 mM imidazole, lysozyme, DNase, and Pierce protease inhibitor cocktail). The cells were lysed by sonication at 60 mA, 10 seconds on followed by 10 seconds off, for a total of 2 minutes. The cell debris was pelleted at 13,000 rpm for 40 minutes. The soluble cell extracts were incubated with Ni-NTA agarose resin (Thermo) according to manufactures protocol. Beads were washed with 20 column volumes of was buffer (50 mM Tris-HCl pH 8.0, 200 mM NaCl, 10% glycerol 5 mM β-ME, and 25 mM imidazole). Proteins were eluted with 5 column volumes of elution buffer (50 mM Tris pH 8.0, 200 mM NaCl, 5 mM β-ME, 10% glycerol and 400 mM Imidazole). Proteins were next purified via size exclusion chromatography on AKTA pure FPLC system with 50 mM Tris pH 8.0, 200 mM NaCl, and 10% glycerol. Fractions with pure protein were combined and concentrated using Sartorius Vivaspin 10 kDa centrifugal concentrator. Bradford Dye (BioRad) was used to quantify the protein concentration using BSA as a standard. The concentrated proteins were aliquoted, flash frozen, and stored at -80°C until use.

4. Mutagenesis, expression, and purification of AzF mutants of KDM4A-E

KDM4A-E variants carrying amber suppressor mutation (TAG) at specific sites were generated using the QuikChange Lightning site-directed mutagenesis kit (Agilent Technologies). The resulting mutant plasmids were confirmed by Sanger sequencing. To express variants, BL21 Star (DE3) cells were co-transformed with pEVOL-based *M. jannaschii* TyrRS-$tRNA_{CUA}^{Tyr}$ pair for AzF (Addgene #31186).[9] Cells were recovered for 1-2 hours in 200 μL SOC medium in a 37°C

shaker at 225 rpm prior to plating on an LB Miller agar plate containing 35 μg/mL chloramphenicol, and either 50 μg/mL kanamycin or 100 μg/mL ampicillin depending on the KDM vector antibiotic resistance. Single colonies were picked and added to each of four inoculates containing 10 mL of Luria-Bertani (LB) Miller broth in presence of appropriate antibiotics. Overnight cultures were centrifuged (ThermoScientific Sorvall Legend XTR Centrifuge, TX-1000 rotor, 4°C) for 10 min at 1000 x g, 2100 rpm. Supernatant was removed, and cell pellets were resuspended in ~1mL M9 media and used to inoculate 1L of GMML medium (M9 minimal media supplemented with 1% v/v glycerol, 300 μM leucine, 1 mM $MgSO_4$, 0.1 mM $CaCl_2$, appropriate antibiotics, and trace amounts of Na_2MoO_4, $CoCl_2$, $CuSO_4$, $MnSO_4$, $MgSO_4$, $FeCl_2$, $CaCl_2$, and H_3BO_3). Cells were allowed to grow at 37 °C to an optical density (OD600) of 0.8. AzF was prepared by diluting in 20mL sterilized deionized water and added aseptically to a final concentration of 1mM. Cells were allowed to shake an additional 30 minutes at 17°C, at which time the synthetase expression was induced with 0.05% w/v arabinose and allowed to shake an additional 30 minutes at 17°C. Finally, 0.6-1.0 mM IPTG was added to induce KDM expression while shaking overnight at 17°C with an Innova 44® Incubator shaker (New Brunswick Scientific). Protein purification, concentration, and storage were performed as described above for the wild type proteins.

5. Expression and purification of histone H3 and its mutants

Gene sequence encoding wild type *Xenopus laevis* histone H3 was a kind gift from Prof. Minkui Luo at the Memorial Sloan-Kettering Cancer Center. Protein expression and purification were carried out as reported earlier.[4] The plasmid containing histone H3-C110A was transformed into BL21 codon plus (DE3) RIPL competent cells. A single colony was picked up and grown overnight at 37°C in 10 mL of LB broth with 100 μg/mL ampicillin and 35 μg/mL chloramphenicol. The inoculation culture was diluted 1:100 fold in fresh LB medium and cells were grown at 37°C until OD_{600} reached to ~0.7. Protein expression was induced by the addition of 0.3 mM IPTG followed by growing for an additional 3 h at 37°C. Cells were harvested by centrifugation at 5000 rpm for 30 min, and then resuspension of the pellet in 5 mL of lysis buffer (10 mM Tris-HCl pH 7.5, 2 M guanidinium hydrochloride (Gdn.HCl), 5 mM *β*-mercaptoethanol, 10% glycerol, DNase, Lysozyme and Roche protease inhibitor cocktail). The cells were lysed by pulsed sonication and centrifuged at 20,000g for 40 min at 4°C. Insoluble histone was recovered from inclusion bodies

by dissolving in 6 M Gdn.HCl and 10 mM Tris-HCl pH 7.5, and incubated for 10 min at room temperature followed by centrifugation at 20,000g for 40 min at 4°C. The soluble histone supernatant was purified by size exclusion chromatography on a Superdex-200 using AKTA pure FPLC system.[5] Fractions were concentrated using Amicon Ultra-4 centrifugal 3K filter and further purified with preparative reverse-phase HPLC (XBridge C18, 5 µm, 10 x 250 mm column) eluting with a flow rate of 4 mL/min starting from 10% acetonitrile to 70 % in 15 min and then to 100 % over 5 min in aqueous trifluoroacetic acid (0.01%). The purified protein was concentrated by SpeedVac followed by lyophilization. The protein was stored at -80°C before use. Histone H3 mutants K4C, K9C, K27C and K36C were generated by the QuikChange Lightening site-directed mutagenesis kit (Agilent Technologies) following manufacturer's protocol. The resulting mutant plasmid was confirmed by DNA sequencing. The mutant proteins were expressed and purified as described above for wild type Histone H3.

6. Chemical trimethylation of H3 mutants (semi-synthetic histones)

For the generation of full-length H3K_C4me3, H3K_C9me3 and H3K_C36me3 proteins, 1 mg of each lyophilized histone mutant (K4C, K9C and K36C) was dissolved in 98 µl of alkylation buffer (4 M Guanidine-Hydrochloride, 1 M HEPES pH 7.8, 10 mM D/L-methionine).[10,11] Once histone was fully dissolved, 2 µl of 1 M DTT (prepared fresh) was added to the histone solution followed by 1 h incubation at 37°C. The solution was then added to 10 mg of (2-bromoethyl) trimethyl ammonium bromide (Sigma, cat# 117196) and protected from light. After incubating for 2.5 h at 50°C (gently agitating every 30 min), 1 µl of 1 M DTT was added to quench the reaction. The solution incubated at 50°C for an additional 2.5 h. The reaction was quenched by adding 5 µl β-mercaptoethanol (β-ME). A PD10 (GE, cat# 17-0851-01) column was used to desalt the solution and the histones were eluted with 2 mM β-ME. SDS-PAGE was performed to determine presence of the histone protein. The samples were lyophilized and then resuspended in 50 mM Tris pH 8.0. Protein concentration was determined by Bradford assay and the molecular weight was verified by LC-MS. Protein was stored in -80°C until used in demethylase activity assay.

7. Expression and purification of lysine methyltransferases and Chromodomains

Catalytic domains of G9a and Suv39H2 and chromodomains of CBX1 were expressed and purified as reported previously.[2] The following N-terminal 6xHis-tagged lysine methyltransferase bacterial

expression constructs in pET28a or pET 28-MHL were obtained from Addgene: CAMKMT (Addgene #58761), DOT1L (Addgene #36196), METTL20 (Addgene #85115), METTL21A (Addgene #60091) and METTL21C (Addgene #60100). Plasmids were transformed into *E. Coli* BL21 (DE3) competent cells (Invitrogen) using appropriate resistant antibiotic. A single colony was picked up and grown overnight at 37 °C in 10 mL of Luria-Bertani (LB) broth in the presence of 50 μg/mL kanamycin and 35 μg/mL chloramphenicol. The culture was diluted 100-fold and allowed to grow at 37°C to an optical density (OD600) of a minimum of 0.8, and protein expression was induced overnight at 17 °C with 0.5 to 1.0 mM IPTG in an Innova 44® Incubator shaker (New Brunswick Scientific). Proteins were purified as follows: harvested cells were resuspended in 15 mL lysis buffer (50 mM Tris-HCl pH 8.0, 200 mM NaCl, 5 mM β-mercaptoethanol, 10% glycerol, 25 mM imidazole, Lysozyme, DNase, and Roche protease inhibitor cocktail). The cells were lysed by pulsed sonication (Qsonica-Q700), and centrifuged at 13,000 rpm for 40 min at 4°C. The soluble extracts were subject to Ni-NTA agarose resin (Thermo) according to manufacturer's instructions. After passing 20 volumes of washing buffer (50 mM Tris-HCl pH 8.0, 200 mM NaCl, 5 mM β-mercaptoethanol, 10% glycerol, and 25 mM imidazole), proteins were eluted with a buffer containing 50 mM Tris-HCl pH 8.0, 200 mM NaCl, 5 mM β-mercaptoethanol, 10% glycerol, and 400 mM imidazole. Proteins were further purified by size exclusion chromatography (Superdex-200) using AKTA pure FPLC system (GE healthcare) with buffer containing 50 mM Tris-HCl pH 8.0, 200 mM NaCl, and 10% glycerol. Purified proteins were concentrated using Pierce™ Protein Concentrator PES, 10K MWCO, 5-20 mL (Thermo Scientific™ 88527). The protein concentration was determined using Bradford assay kit (BioRad Laboratories) with BSA as a standard. The concentrated proteins were stored at -80 °C before use.

8. Demethylase activity of wild type and AzF mutant proteins on peptide substrates

To monitor enzymatic activity, a demethylase activity assay was optimized and then observed through MALDI-TOF.[6] Each demethylase assay sample included 10 μM enzyme (wild type or AzF mutant proteins), 10 μM peptide (histone or nonhistone), 1 mM 2OG, 50 mM Tris pH 8, 50 μM $(NH_4)_2Fe(SO_4)_2$, 2OG and 2 mM L-ascorbic acid with a total assay volume of 10 μl. Fe(II) supplement and L-ascorbic acid were prepared freshly. The 2OG was added last to the assay sample and briefly centrifuged. The samples were incubated at 37°C for three hours. To observe demethylase activity, 0.8 μL of sample and 0.8μL of α-cyano-4-hydroxycinnamic acid (CHCA)

(dissolved in 50:50 0.1% TFA and acetonitrile) were combined on a MALDI plate. Samples were ionized using reflectron positive mode on Bruker Daltonics UltrafleXtreme MALDI TOF with detection carried out from 700-3500 m/z. Bruker Flex Analysis software was used to analyze the MALDI-TOF MS spectra.

9. Demethylase activity of AzF mutants on semi-synthetic histones

A demethylase activity assay was conducted to determine if KDM4A-I71AzF mutant was active towards semi-synthetic histones (H3K$_C$4me3, H3K$_C$9me3, H3K$_C$36me3). The samples included 10 μM histone, 10 μM enzyme, 1 mM 2OG, 100 μM $(NH_4)_2Fe(SO_4)_2$, 2 mM L-ascorbic acid, 50 mM Tris pH 8.0 (degassed), 1 mM TCEP (freshly prepared) and 10% glycerol (v/v). The samples were incubated for 3 h at 37°C and were then purified through a C-18 Sep-pak column (Waters, cat# WAT054955). The histones were concentrated by speed vacuum and analyzed for demethylation by ESI LC-MS. The negative control did not include the mutant enzyme.

10. Photo-crosslinking experiment with peptides and in-gel fluorescence

For peptide photo-crosslinking experiments, 10 μM TAMRA-labeled H3K9me0 or H3K9me3 peptide was mixed with 20 μM of wild type KDM4A or AzF variants in a buffer consisting of 2 mM ascorbic acid, 50 mM tris pH 8.0, 50 μM $Fe(NH_4)_2(SO_4)_2$, 200 μM 2OG. Samples were exposed to UV lamp (MAXIMA ML-3500S UV, 50,000 μW/cm^2, 350nm-385nm 50% irradiated, 365nm 100% irradiated) for 30 minutes at 4°C on ice. Negative controls were kept in dark on ice for the full 30 minutes. 10 μL of sample was mixed with 5 μL 4X Laemmli dye (Biorad #1610747) and separated on a 4-12 % Criterion XT precast gel (Bio-Rad Laboratories). Gels were imaged on a ChemiDoc MP Imaging system using TAMRA fluorophore excitation wavelength (Emission filter 605/50, Light: green Epi illumination). The gel was subsequently stained with Coomassie brilliant blue R-250 staining solution to confirm the presence of proteins in all the samples.

11. Photo-crosslinking experiment with semi-synthetic and extracted histones

5 μg of synthetic histone (H3K$_C$4me3 or H3K$_C$9me3 or H3K$_C$36cme3) or 5 μg of extracted histones was incubated with 20 μM of AzF mutants in the binding buffer (10 mM Tris-HCl pH 7.5, 150 mM NaCl, 0.05% Tween 20, and 0.5 mM TCEP). The samples were subjected to UV irradiation at 365 nm for 30 min at 4°C. Negative controls were kept in the dark. The samples were

separated on 15% SDS-PAGE gel and onto 0.2 μm nitrocellulose membrane at a constant voltage of 15V for 30 minutes at room temperature. The membrane was washed three times in TBST buffer (50 mM Tris pH 7.4, 200 mM NaCl, and 0.01% Tween) for 5 mins at room temperature and blocked for an hour at room temperature in in 5% BSA buffer prepared in TBST. Immunoblotting was performed with primary antibodies with dilutions as per manufacturer's protocol for Anti-6X His tag® antibody - ChIP Grade (cat #ab9108, Abcam) and H3 C-term (Invitrogen cat# 701517) overnight at 4°C. The membranes were washed with TBST buffer thrice at room temperature for five minutes each. The blots were then incubated with the HRP-conjugated secondary antibodies Goat anti-Rabbit IgG (Active Motif cat# 15015) with 5% BSA in TBST. The membranes were washed again with TBST buffer thrice at RT for five minutes each. Protein bands were visualized by chemiluminescence using VISIGLO HRP Chemiluminescent substrates A and B (cat# N252-120ML and N253-120ML, aMReSCO) following manufacturer's protocol.

12. Preparation of mammalian cell extracts for photo-crosslinking

Human embryonic kidney (HEK) 293T cells were grown in Dulbecco modified Eagle medium (DMEM) (Gibco) supplemented with 10% fetal calf serum in a humidified atmosphere containing 5% CO_2 in a T75 flask. At ~80% confluence, cells were treated with 20 μM of KDM4 inhibitor n-octyl-IOX1 (EMD Millipore)[12,13] dissolved in DMSO to generate hypermethylated proteome. 24 hours post treatment, cells were harvested and lysed with 300 μL of cold RIPA buffer (Sigma) supplemented with 1X Roche protease inhibitor cocktail and 5 mM TCEP by sonicating for 15 min at amplitude of 100 with a repeating 1 min pulse cycle at 4°C. Cell lysates were centrifuged at 12,000 rpm for 30 min at 4 °C to remove cell debris. The supernatant was then passed through the detergent removal spin column (Pierce # 87778) and eluted with Tris buffer (50 mM Tris-HCl pH 8.0, 10% glycerol, 2 mM TCEP, 1X Roche protease inhibitor cocktail) following manufacturer's protocol. Protein concentration was determined by Bradford assay (Bio-Rad Laboratories). This stock solution was used for photo-crosslinking experiment as described below.

13. Photo-crosslinking with HEK293T cell extracts

For photo-crosslinking studies, 1.0 mg of n-octyl-IOX1 treated HEK293T cell lysates was incubated with 50 μM of KDM4A-I71AzF mutant carrying an N-terminus Strep tag of sequence

WSHPQFEK in a buffer containing 10 mM Tris-HCl pH 7.5, 150 mM NaCl, 0.05% Tween 20, and 0.5 mM TCEP. The samples were subjected to UV irradiation at 365 nm for 1 hr. at 4°C. Negative controls were not subjected to UV exposure. Samples were then bound to StrepTactin resin (Qiagen, cat# 30004) for 1 hr. at 4°C with gentle rotation. To remove un-crosslinked cellular proteins, samples were washed with buffer (50 mM Tris-HCl pH 8.0, 200 mM NaCl, 5 mM β-mercaptoethanol, 10% glycerol) three times and the bound proteins were eluted with a buffer containing 50 mM Tris-HCl pH 8.0, 200 mM NaCl, 5 mM β-mercaptoethanol, 10% glycerol, and 5 mM D-desthiobiotin. The eluted proteins were separated on a 4-12% Criterion XT precast SDS-PAGE gel (Bio-Rad Laboratories) and analyzed by Coomassie staining, Western blotting and tandem mass spectrometry.

14. LC-MS analysis of the intact H3 and KDM4 proteins

Sample separation was performed by a Thermo Scientific Dionex UltiMate 3000 UHPLC+ with a Thermo ProSwift RP-2H analytical 4.6x50 mm SS column with flow rate 0.2 mL/min of a binary solvent system (Solvent A: 0.1% Formic Acid in HPLC grade H_2O, Solvent B: 0.1% Formic Acid in HPLC grade acetonitrile). A multistep gradient was employed depending on the nature of the protein. A typical method includes 10%B at t=0 minute followed by increasing to 50% over 21 minutes, then to 80% at 22 minutes, and subsequently equilibrating back to 10%B. Samples were ionized using electrospray ionization in positive mode and ions were detected in Thermo Scientific Q-exactive Orbitrap. Chromatogram peak width was 10 seconds, resolution was 17,500, microscans was 5, maximum IT was 250 s, scan range was 500-3000 m/z, and automatic gain control (AGC) target was 3 x 10^6. Data was analyzed using Thermo Scientific Protein Deconvolution 3.0 software via Manual ReSpect (isotopically unresolved) method (chromatogram parameters: high sensitivity, auto spectral averaging False) (main parameters:, negative charge false, charge carrier H^+ (1.00727663), m/z range 1000-3000, output range 10000-25000, mass tolerance 5 ppm, charge state range 10-100, calculate XIC True, Peak Model Intact Protein) (advanced parameters: minimum peak significance 1 standard deviation, Noise Rejection 95% Confidence, Use relative Intensities True, number of iterations 3, minimum adjacent charges 6-10, number of peak modes 1, resolution at 400m/z 12374, and left/right peak shape 2:2).

15. LC-MS/MS analysis of the affinity-purified proteins

In gel trypsin digestion. In-gel trypsin digestion was carried out as previously described.[14] Excised gel bands were washed with HPLC water and de-stained with 50% acetonitrile (ACN)/25mM ammonium bicarbonate until no visible staining. Gel pieces were dehydrated with 100% ACN, reduced with 10mM dithiothreitol (DTT) at 56°C for 1 hour, followed by alkylation with 55mM iodoacetamide (IAA) at room temperature for 45min in the dark. Gel pieces were then again dehydrated with 100% ACN to remove excess DTT and IAA, and rehydrated with 20ng/µl trypsin/25mM ammonium bicarbonate and digested overnight at 37°C. The resultant tryptic peptides were extracted with 70% ACN/5% formic acid, vacuum dried and re-constituted in18µl 0.1% formic acid.

Tandem mass spectrometry. Proteolytic peptides from in gel trypsin digestion were analyzed by a nanoflow reverse-phased liquid chromatography tandem mass spectrometry (LC-MS/MS). Tryptic peptides were loaded onto a C18 column (PicoChip™ column packed with 10.5cm Reprosil C18 3µm120Å chromatography media with a 75µm ID column and a 15µm tip, New Objective, Inc., Woburn, MA) using a Dionex HPLC system (Dionex Ultimate 3000, ThermoFisher Scientific, San Jose, CA) operated with a double-split system to provide an in-column nano-flow rate (~300nl/min). Mobile phases used were 0.1% formic acid for A and 0.1% formic acid in acetonitrile for B. Peptides were eluted off the column using a 52-minute gradient (2-40% B in 42 min, 40-95% B in 1min, 95% B for 1 min, 2% B for 8 min) and injected into a linear ion trap MS (LTQ-XL, ThermoFisher Scientific) through electrospray.

The LTQ XL was operated in a date-dependent MS/MS mode in which each full MS spectrum [acquired at 30000 automatic gain control (AGC) target, 50ms maximum ion accumulation time, precursor ion selection range of m/z 300 to1800] was followed by MS/MS scans of the 5 most abundant molecular ions determined from full MS scan (acquired based on the setting of 1000 signal threshold, 10000 AGC target, 100ms maximum accumulation time, 2.0 Da isolation width, 30ms activation time and 35% normalized collision energy). Dynamic exclusion was enabled to minimize redundant selection of peptides previously selected for CID.

Peptide identification by database search. MS/MS spectra were searched using MASCOT search engine (Version 2.4.0, Matrix Science Ltd) against the UniProt human proteome database. The following modifications were used: static modification of cysteine (carboxyamidomethylation, +57.05 Da), variable modification of methionine (oxidation, +15.99 Da). The mass tolerance was

set at 1.4 Da for the precursor ions and 0.8 Da for the fragment ions. Peptide identifications were filtered using PeptideProphet™ and ProteinProphet® algorithms with a protein threshold cutoff of 99% and peptide threshold cutoff of 90% implemented in Scaffold™ (Proteome Software, Portland, Oregon, USA). The analyzed proteomic data are provided in Table S2.

16. Methylation activity of wild type KMTs on eIF4A3-K374me0/1/2 peptides

20 µM lysine methyltransferase, 10 µM eIF4A3-me0/1/2 peptide and 200 µM SAM were incubated at room temperature for 1-2 hours in 50 mM Tris pH 8.0. Reaction was stopped by storing the microcentrifuge tubes on ice. After mixing, 0.8 µL of sample and 0.8 µL of α-cyano-4-hydroxycinnamic acid (CHCA) (dissolved in 50:50 0.1% TFA and acetonitrile) were combined on a MALDI plate. Samples were ionized using reflectron positive mode on Bruker Daltonics UltrafleXtreme MALDI TOF with detection carried out from 700-3500 m/z. Bruker Flex Analysis software was used to analyze the MALDI-TOF MS spectra.

17. Isothermal Titration Calorimetry

Isothermal titration calorimetry (ITC) was performed with an ITC200 instrument (MicroCal, Malvern). Experiments were conducted at 25 °C while stirring at 750 rpm. Buffers of protein (CBX1) and peptides (eIF4A3-Kme0 **21**, eIF4A3-Kme1 **22**, eIF4A3-Kme2 **22**, eIF4A3-Kme3 **13**) were matched to 200mM NaCl, 10% glycerol, and 50mM Tris pH 8.0. Each titration was performed as follows: one initial injection of 0.4 µL for 0.8 seconds, followed by 19 injections of 2.0 µL for 4 seconds, with ≥2 mins between each injection. The initial injection was discarded prior to data analysis. The microsyringe (40µL) was loaded with 1.1 mM peptide and injected into the cell (200µL), occupied by CBX1 chromodomain at a concentration of 110 µM. Data was fitted to a single binding site model using Microcal ITC200 Software with Origin Lab 7.

18. Expression of full-length KDM4A and G9a in HEK293T cells

KDM4A mammalian expression plasmid in CMV vector was purchased from Addgene (plasmid #24180). Full-length G9a in pCDNA3 vector was kind from Prof. Minkui Luo at the Memorial Sloan-Kettering Cancer Center. HEK293T/17 cells (ATCC) were grown in a T25 containing Delbucco's Modified Eagle Medium (DMEM) (Corning) with 10% FBS at 37°C, 5% CO_2, and 95% relative humidity. When cells reached 80% confluency, 5 µg of plasmid and 10 µg

lipofectamine 2000 (Invitrogen) were incubated with 500 μL Optimem (Gibco), after 5 minutes the plasmid and lipofectamine were combined and incubated at room temperature for 20 minutes. The media was changed and the 1 mL of Optimem was added to the flask. Cells were returned to the incubator to grow for 24 hours. The media was removed, and cells were washed with ice cold PBS (Corning), then trypsinized with TrpLE Express (Gibco). The trypsinization reaction was quenched with DMEM 10% FBS and cells were pelleted then washed once with ice cold PBS. The nuclear extracts were generated by first resuspending HEK293T cell pellets in 700 μL nuclear isolation buffer (15 mM Tris pH 7.5, 60 mM KCl, 15 mM NaCl, 5 mM $MgCl_2$, 1 mM $CaCl_2$, 1mm DTT, 2 mM Na_3VO_4, 250 mM sucrose, 1X Pierce protease inhibitor (Thermo Fisher #PIA32955), 1 mM PMSF, 0.3% NP40 diluted to volume with MilliQ H_2O) and incubated on ice for 15 minutes. The samples were then pelleted by centrifugation at 2000 rcf for 5 minutes. The supernatant was removed and the pelleted nuclei were resuspended in 200 μL Pierce IP lysis buffer (Thermo Fisher #87787) containing 1X Pierce protease inhibitor and left on ice for 5 minutes. The nuclei were then sonicated at 100% amp for 5 minutes (pulsed 1 minute "on", 20 seconds "rest") using a Qsonica-Q700, cuphorn sonicator. The samples were pelleted once again at 2000 rcf for 5 minutes and the supernatant containing the nuclear extracts were collected. $MgCl_2$ (1M) solution was added to each sample give a final concentration of 6 mM. The nuclear extracts were quantified with Bradford assay (Bio-Rad laboratories) using BSA as a standard and used for immunoprecipitation and pull-down experiments as described below.

19. Knock down of KDM4A using siRNA

HEK293T cells were plated in T75 flasks in DMEM 24 hours prior to transfection. Lipofectamine 3000 (Thermo Fisher Cat # L3000015) was used to transfect cells with 4 μg Silencer™ Negative Control No. 1 siRNA (Thermo Fisher Cat #4390843), 4 μg Silencer KDM4A siRNA s18636 (Thermo Fisher Cat # AM16708), or 15 μg full length KDM4A (Addgene plasmid #24180). The siRNAs were selected based on their reported efficacy to silence KDM4A in human cells (*Cancer Discov*. **2015**, *5*, 255-63).[15] The complexes were incubated in 1.5 mL of Opti-MEM (Thermo Fisher Cat# 31985070) for 20 minutes before being added to each flask. Media was refreshed after 6 hours. After an additional 42 hours, the media was replaced once more. Cells were collected 72 hours post transfection. Nuclear extracts were generated from each sample and used for SDS PAGE followed by Western blot.

20. Fixed cell immunofluorescence imaging

Coverslips (Fisher #12-545-80P) were treated with poly-d-lysine (Fisher A3890401) and washed 3 times with PBS and left to dry. Coverslips were placed in a 24-well plate and HEK293T cells were cultured on each coverslip. After 36 hours, the cells were washed 3x with ice cold PBS pH 7.4 and fixed at room temperature using 2% paraformaldehyde in PBS pH 7.4 for 20 minutes. The cells were then washed 3 more times with PBS before permeabilizing with 0.2% triton X in PBS pH 7.4 for 25 minutes. Cells were washed 3x in PBB (0.5% BSA in PBS pH 7.4). A blocking solution of 5% BSA in PBS pH 7.4 was added to the cells and left to incubate at room temperature for 45 minutes. The cells were washed 3 times with PBB. Primary antibody solutions were formulated with 1:100 dilutions of the primary antibody (1:500 CBX1) in PBB and added to the cells for 1.5 hours in the dark, at room temperature. Primaries used include CBX1 cat#8676S CST, eIF4A3-AlexaFluor 488 cat#sc-365549 AF488 Santa Cruz Biotechnology, KDM4A mAb cat#5328 CST, and G9a cat#3306S CST. The cells were washed 3x with PBB and then incubated with a secondary antibody (1:1000 Cy3 Goat anti-Rabbit Fisher #A10520) for 1 hour, in the dark, at room temperature. The cells were washed 3x with PBS pH 7.4 and then mounted onto a glass slide with (ProLong Diamond Antifade mountant with DAPI Themo #P36962). Coverslips were left to dry overnight and were imaged on an Olympus Fluoview 1000 confocal microscope with a 60X objective lens with 1.5X zoom. Scale bars were added using NIS-Elements software.

21. Interactome pull-down using recombinant CBX 1 protein

A 50 µL slurry of Thermo Scientific™ HisPur™ Ni-NTA Magnetic Beads (Thermo Fisher #88831) was aliquoted for each treatment sample. They were equilibrated in binding buffer (50 mM Tris pH 8.0, 200 mM NaCl, and 10% glycerol) before adding 150 µg of recombinant CBX1 chromodomain containing a 6xHis tag. The protein and beads were gently mixed at 4°C for 30 minutes. Following that, 1800 µg of nuclear extracts were added to each microcentrifuge tube containing the CBX1 bound beads. The samples were left to mix on a carousel for 2 hours at 4°C. The beads were pelleted using a magnetic microcentrifuge rack and the supernatant was discarded. They were then washed twice with 50 mM Tris pH 7.5, 100 mM KCl, 0.1% Triton X. Lastly, the proteins were eluted with a buffer comprised of 50 mM Tris pH 7.5, 50 mM NaCl, 500 mM imidazole, and 5 mM β-mercapoethanol. Laemmli dye (4X) was added to achieve a final 1X

concentration. The samples were then heated to 65°C for 15 minutes and loaded into a 4-12% polyacrylamide gel for Western blotting.

22. Immunoprecipitation using eIF4A3 and CBX1 antibodies

With eIF4A antibody: Protein A Dynabeads (Fisher kit cat#10006D) were mixed into a slurry and dispensed into microcentrifuge tubes in 80 µl aliquots. Using a magnetic microcentrifuge rack to pellet the beads, the supernatant was removed and the beads were washed twice with 200 µl of the corresponding kit's "binding and wash buffer". Each microcentrifuge tube received an eIF4A3 antibody mixture containing 45 µL eIF4A3 (#sc-365549 Santa Cruz Biotechnology) and 2.5 µL eIF4A3 (#PA5117930 Thermo Fisher). The antibodies were incubated with the beads using gentle agitation for 1 hour at 4°C. The beads were then washed 2x with conjugation buffer (200 µl of 20 mM sodium phosphate, 0.15M NaCl). BS^3 crosslinking solution (250 µl of 5 mM BS^3 in conjugation buffer) was added to each aliquot and incubated at room temperature for 20 minutes. The supernatant was removed and a second round of BS^3 solution was administered for 5 minutes at room temperature. The reaction was quenched with 12.5 µL of 1M Tris pH 7.5 and left to incubate for 15 minutes at room temperature before discarding the supernatant. To remove any uncrosslinked antibody, the beads were washed with 250 µL 1M glycine pH 3 for 10 minutes. To equilibrate the beads for the nuclear extracts, they were washed twice with 200 µL Pierce IP lysis buffer (Thermo Fisher #87787). Then 4 mg of nuclear extracts were added and left mixing overnight at 4°C. The following day, the beads were pelleted, and the supernatant was removed. The beads were washed twice with 50 mM Tris pH 7.5, 100 mM KCl, 0.1% Triton X. Laemmli dye (4X) was added to "elution buffer" from the Protein A Dynabead kit for a final 1X concentration. Each sample received 60 µL of the denaturing elution solution and was then incubated at 65°C for 15 minutes. The beads were pelleted on the magnetic rack and the supernatants were collected to perform Western blots.

With CBX1 antibody: Protein A Dynabeads (Fisher kit cat#10006D) were mixed into a slurry and dispensed into microcentrifuge tubes in 80 µl aliquots. Using a magnetic microcentrifuge rack to pellet the beads, the supernatant was removed, and the beads were washed twice with 200 µl of the corresponding kit's "binding and wash buffer". Each microcentrifuge tube received an CBX1 antibody mixture containing 10 µL antibody (ProteinTech cat. # 10241-2-AP). The antibodies

were incubated with the beads using gentle agitation for 1 hour at 4°C. The beads were then washed 2x with conjugation buffer (200 µl of 20 mM sodium phosphate, 0.15M NaCl). BS^3 crosslinking solution (250 µl of 5 mM BS^3 in conjugation buffer) was added to each aliquot and incubated at room temperature for 20 minutes. The supernatant was removed and a second round of BS^3 solution was administered for 5 minutes at room temperature. The reaction was quenched with 12.5 µL of 1M Tris pH 7.5 and left to incubate for 15 minutes at room temperature before discarding the supernatant. To remove any uncrosslinked antibody, the beads were washed with 250 µL 1M glycine pH 3 for 10 minutes. To equilibrate the beads for the nuclear extracts, they were washed twice with 200 µL Pierce IP lysis buffer (Thermo Fisher #87787). Then 4 mg of nuclear extracts were added and left mixing overnight at 4°C. The following day, the beads were pelleted, and the supernatant was removed. The beads were washed twice with 50 mM Tris pH 7.5, 100 mM KCl, 0.1% Triton X. Laemmli dye (4X) was added to "elution buffer" from the Protein A Dynabead kit for a final 1X concentration. Each sample received 60 µL of the denaturing elution solution and was then incubated at 65°C for 15 minutes. The beads were pelleted on the magnetic rack and the supernatants were collected to perform Western blots.

23. General protocol for Western blotting

Assay samples were mixed with appropriate volume of 4X Laemmli Dye (Bio-Rad), heated at 95°C for 5 minutes and loaded onto 4–12% Criterion™ XT Bis-Tris protein gels (Bio-Rad #3450123) or in-house prepared SDS-polyacrylamide gel and subjected to electrophoresis (Criterion Precast Tank, Bio-Rad 1656001) typically at 150V for 30 min - 1 hour in 1X MES buffer (prepared from Invitrogen™ Novex™ 20X Bolt™ MES SDS Running Buffer). After electrophoresis, gels were removed from cassette and transferred onto a 0.2 µm supported nitrocellulose membrane via wet blotting tank at a constant voltage of 80 V for 1 hr. at 4°C or at 40 for 2.5 hr. at 4°C. Occasionally, 0.45 µm nitrocellulose membrane (BIORAD cat #1620112) or 0.2 µm PVDF (Immobilon-PSQ ISEQ00010) membrane preactivated with methanol for 30 seconds was also used. For semidry transfer, gels were incubated for 5 minutes with semidry transfer buffer (48mM Tris Base, 39 mM glycine, 0.0375% SDS, 20% methanol) followed by transfer via semidry blotting apparatus (Bio-Rad #1703940) at a constant voltage of 15 V for 30 minutes or at a constant amplitude of 5.5 mA per square centimeter for 30 minutes with a maximum voltage of 25 V. After transfer, membranes were washed once with TBST (50 mM Tris HCl pH

7.4, 150 mM NaCl, 0.01% Tween-20) prior to blocking with 5% nonfat dry milk or 5% Bovine Serum Albumin (BSA) in TBST buffer for 1 hour at room temperature with gentle shaking. The blocking buffer was removed, and membranes were washed three times with 20 mL of TBST buffer.

Immunoblotting was performed with primary antibodies following recommended dilution (Histone H3 mAb, cat# 61475 from Active Motif or cat# 9715 from Cell Signaling Technology (CST); H3K4me3, cat #9751S; H3K9me3, cat #13969S or H3K9me3 mAb, cat# 61013 from Active Motif; H3K36me3, cat #4909S; pan-trimethyllysine 14680S; pan-dimethyllysine #14117S from CST; Anti-6X His tag® antibody-ChIP Grade, cat #ab9108 from Abcam; H3 C-term. cat# 701517 from Invitrogen, H3 cat#9715S from CST, CBX1 cat# 8676S from CST, CBX3 cat#2619S from CST, eIF4A3 cat#sc-365549 from Santa Cruz Biotechnology, eIF4A3-AlexaFluor 488 cat#sc-365549 AF488, Streptag cat# NBP243719 from Fisher, KDM4A mAb cat#5328 from CST, G9a cat#3306S from CST) at 4°C overnight with gentle shaking. The antibody solutions were removed, and membranes were washed with TBST buffer three times. The blots were then incubated with HRP-conjugated secondary antibody Goat anti-Mouse IgG (cat# 15014, Active motif) with 5% nonfat dry milk (1:10000 dilution) in TBST for 1.5 h at room temperature. After similar washing, protein bands were visualized by chemiluminescence using VISIGLO HRP Chemiluminescent substrates A and B (cat# N252-120ML and N253-120ML, aMReSCO) following manufacturer’s protocol. The antibody solution was removed, and the membranes were washed with TBST 3 times for 5 minutes each, then incubated with 1:5000 dilute HRP-conjugated secondary antibody Goat anti-Rabbit IgG (cat #7040 Cell Signaling Technology) or Goat anti-Mouse IgG (cat #7076 Cell Signaling Technology) for 2 hours at room temperature. To remove the secondary antibodies, the membrane was washed with TBST 3 times, 5 minutes each. Subsequently, the membrane was incubated with Pierce™ ECL Western Blotting Substrate (Thermo Scientific cat # PI32160) following manufacturer’s protocol. For eIF4A3 immunoprecipitation, eIF4A3-AlexaFluor 488 cat#sc-365549 AF488 was used to visualize eIF4A3 levels using fluorescence after primary antibody incubation. All membranes were imaged on BioRad Chemidoc and analyzed with BioRad Image Lab software.

24. Quantification of Western blots and nuclear proteins by Image J

Raw Western blot TIF images were opened in ImageJ software. The first band was selected using a rectangular selection tool. This was designated as the first lane by going to "Analyze", then "Gels", then "Select first lane". Identical rectangular selections were then used to identify the remaining bands from the immunoprecipitated samples. The lanes were plotted, generating peaks from the signal intensities. Each peak was integrated, and the value was normalized to the eIF4A3 signal.

The fixed cells images were opened in ImageJ and converted to 8-bit images. The DAPI signal threshold was set to 34 as a standard. All protein images were set to a threshold of 6. Using the measurement tool, the area and mean signal were determined for each protein. The average value was multiplied by the area to get a total signal for the entire image. To identify the signal in the nucleus, the DAPI signal was used to create a "selection" that was imposed upon the corresponding protein signals. The measurement tool was used again to determine the area and mean signal within the selection. The nuclear average was multiplied by the effective area of the nucleus to get the total nuclear signal. This value was the divided by total signal of the image and multiplied by 100% to determine the percentage of nuclear protein present in the cells. Three replicated were completed, and the average and standard deviation were graphed in GraphPad Prism.

25. Supplementary figures and tables

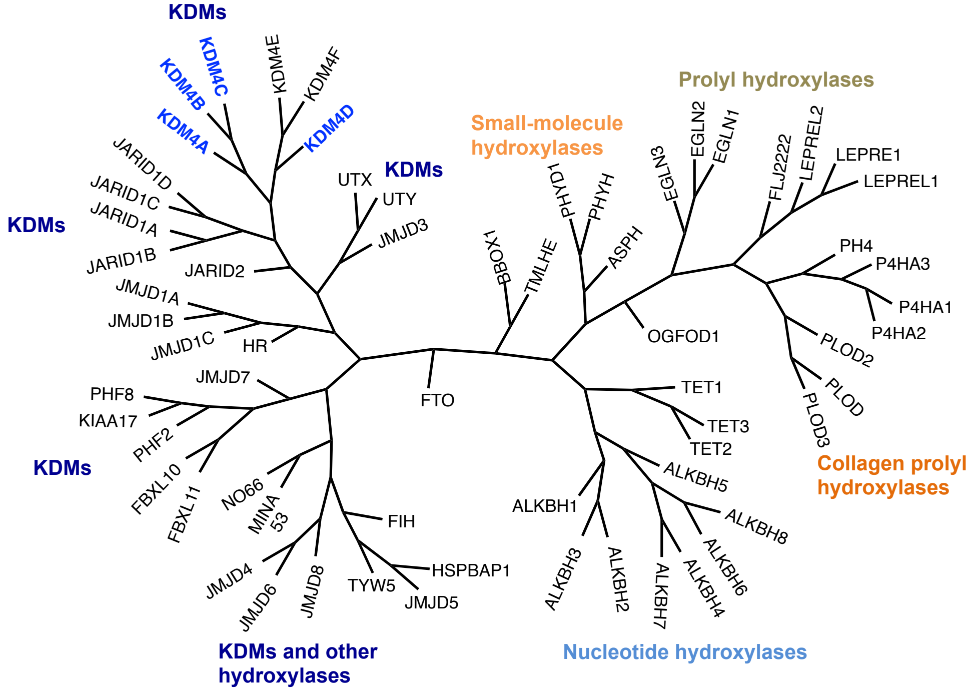

Supplementary Figure S1. Phylogenetic analysis of human 2OG-dependent enzymes including >40 KDMs.[16] Unbiased profiling of substrates of these enzymes has remained underexplored.

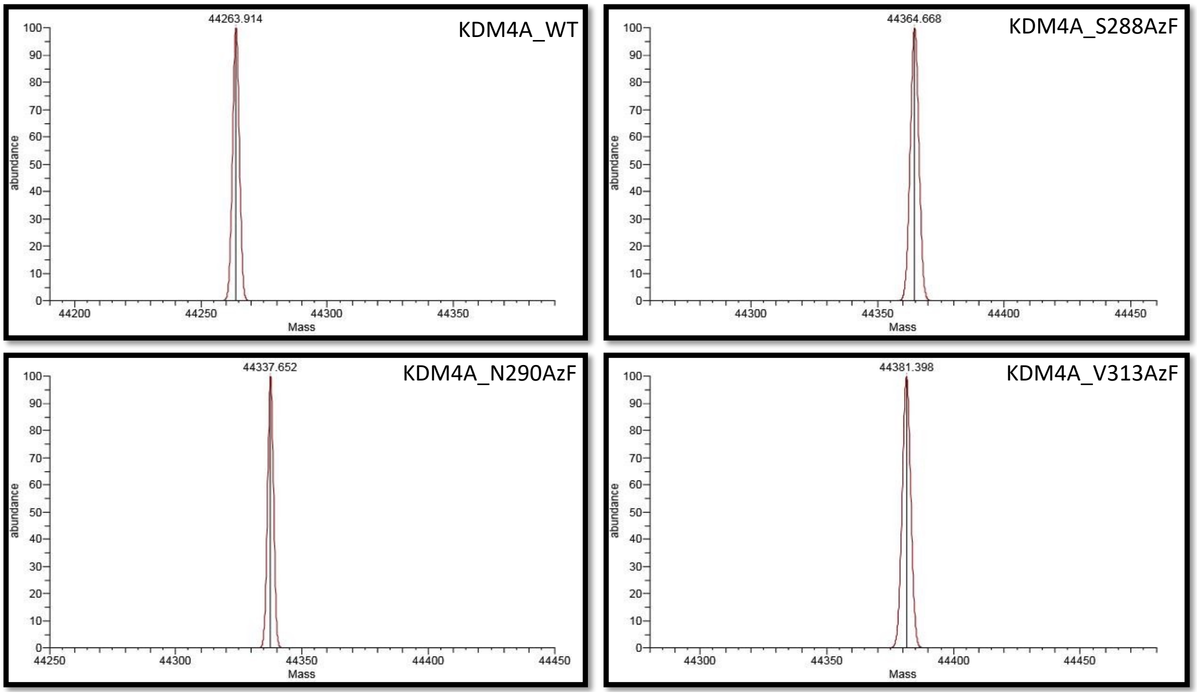

Supplementary Figure S2. Deconvoluted ESI LC-MS spectra of wild type and mutants KDM4s.

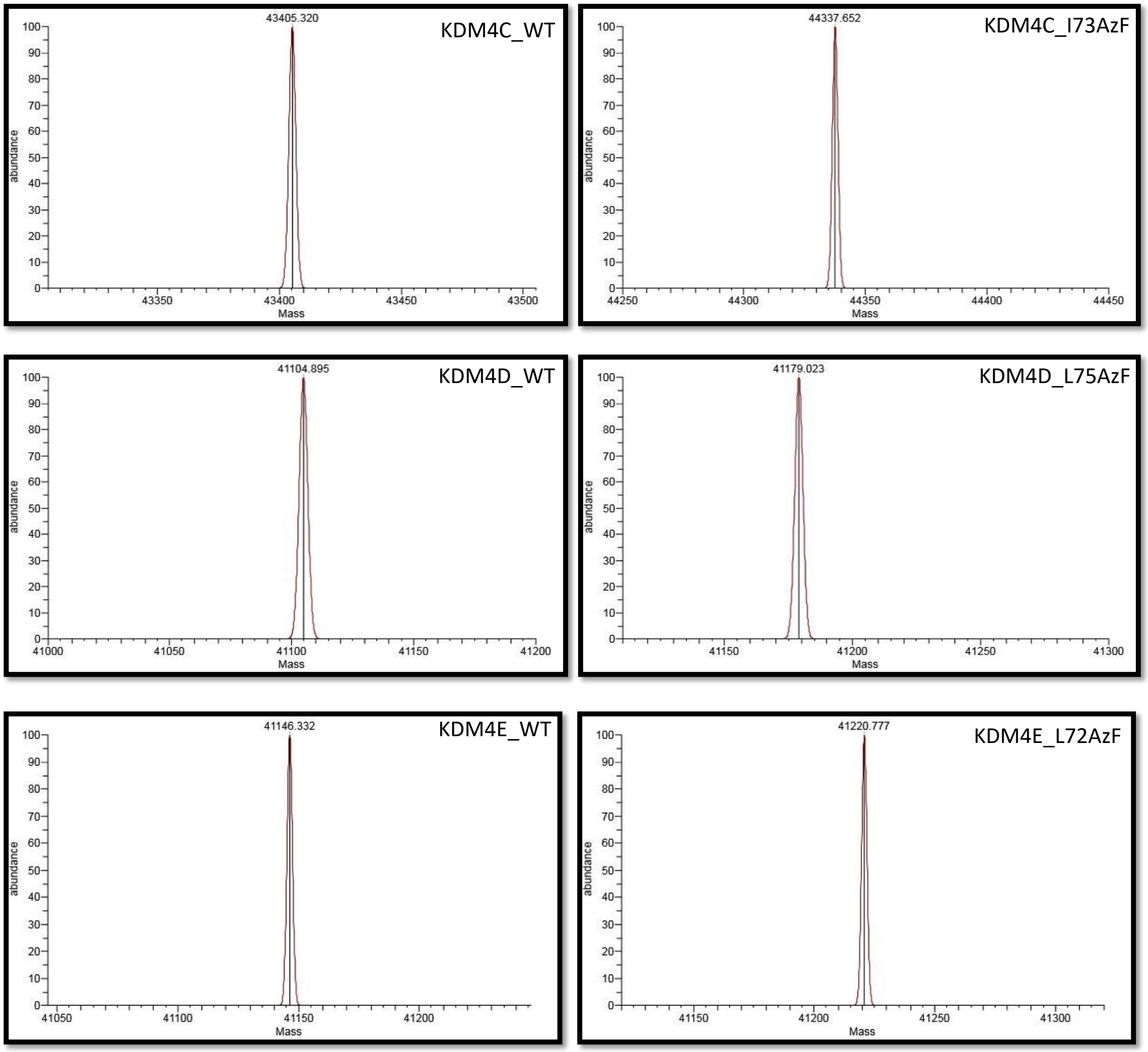

Supplementary Figure S2 cont'd. Deconvoluted ESI LC-MS spectra of wild type and mutants KDM4s.

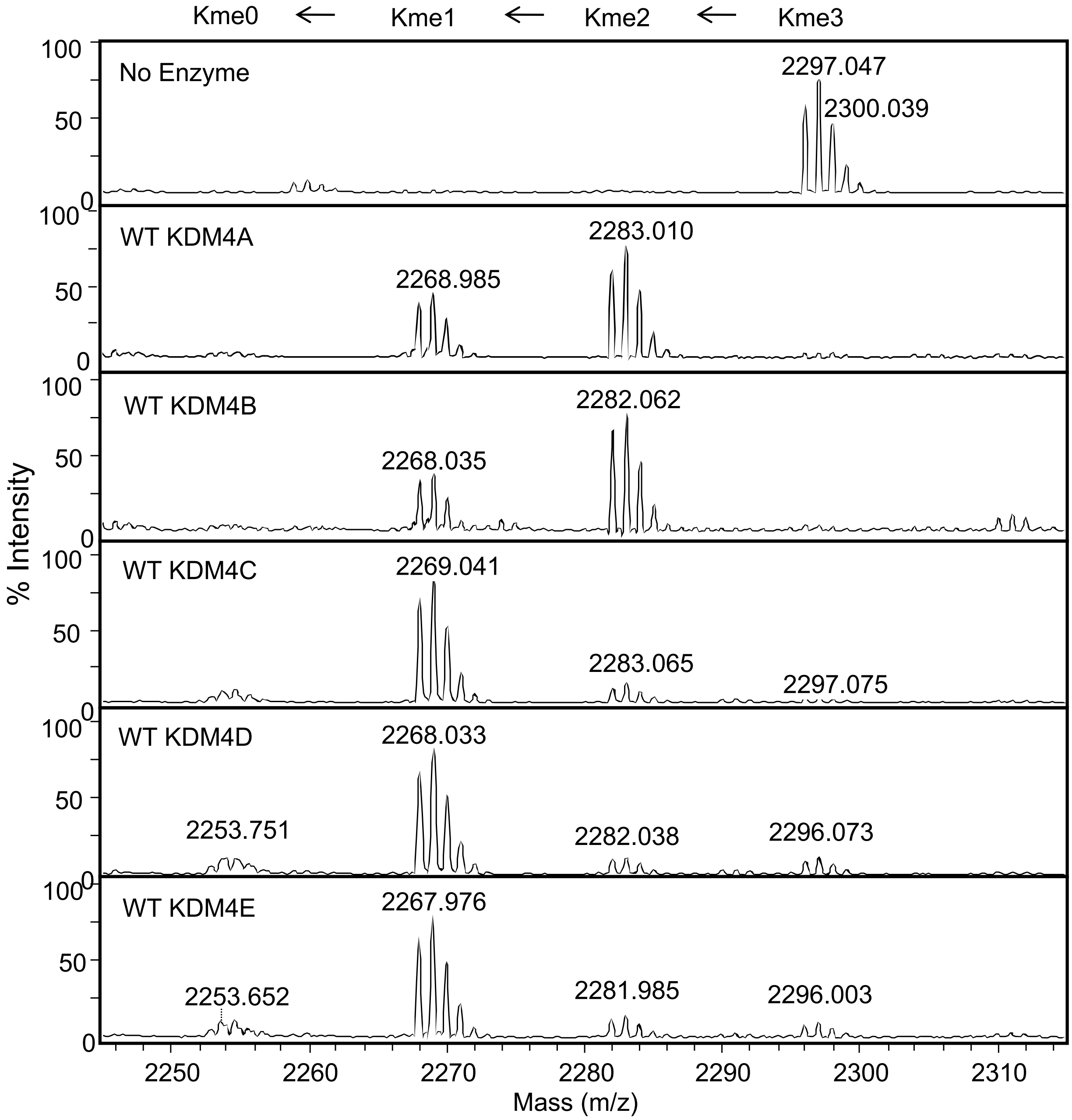

Supplementary Figure S3. MALDI-TOF spectra of activity of wild type KDM4A-E and the AzF mutants towards H3K9me3 peptide **2**.

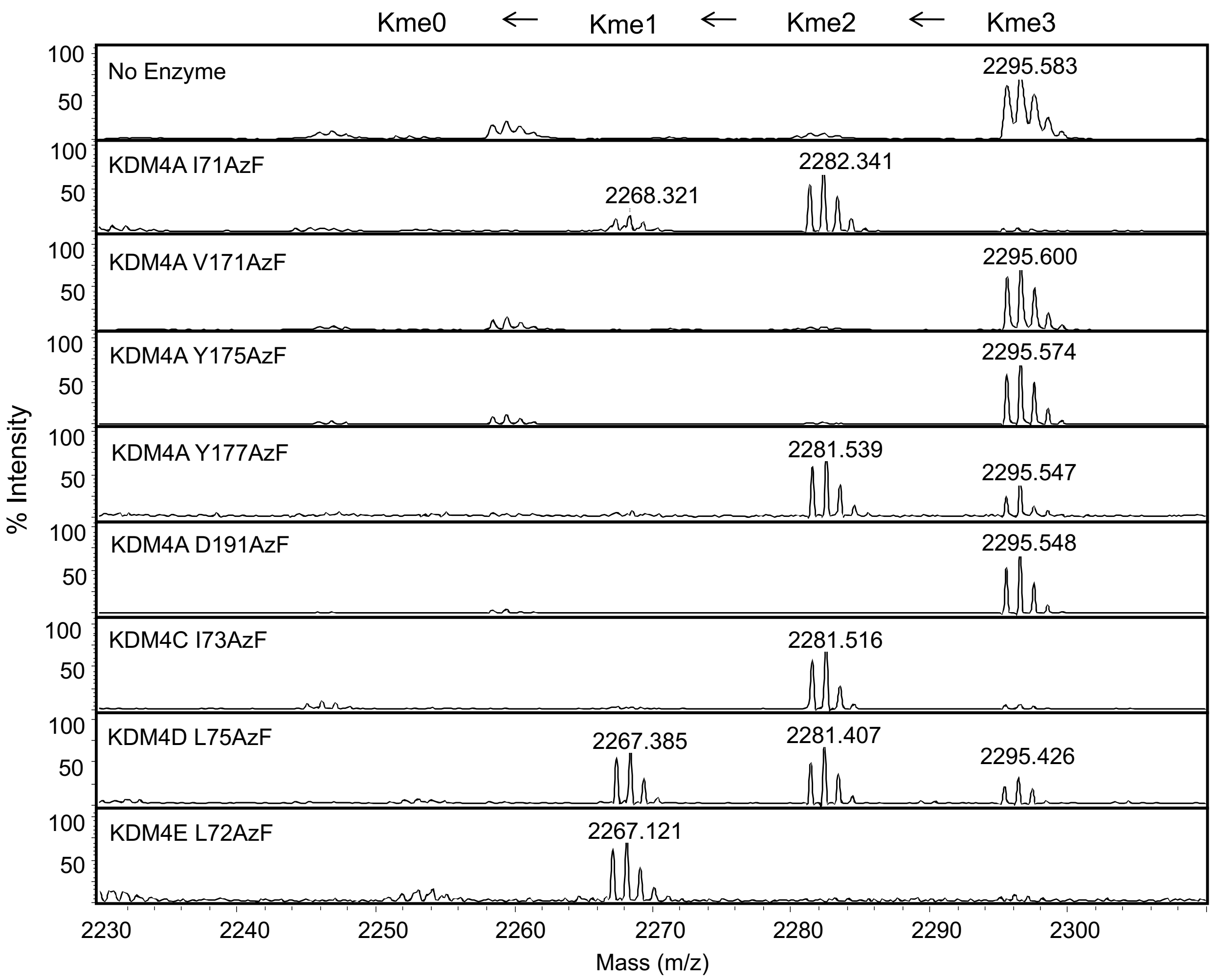

Supplementary Figure S3 Cont'd. MALDI-TOF MS spectra showing activity of wild type KDM4A-E and the AzF mutants towards H3K9me3 peptide **2**.

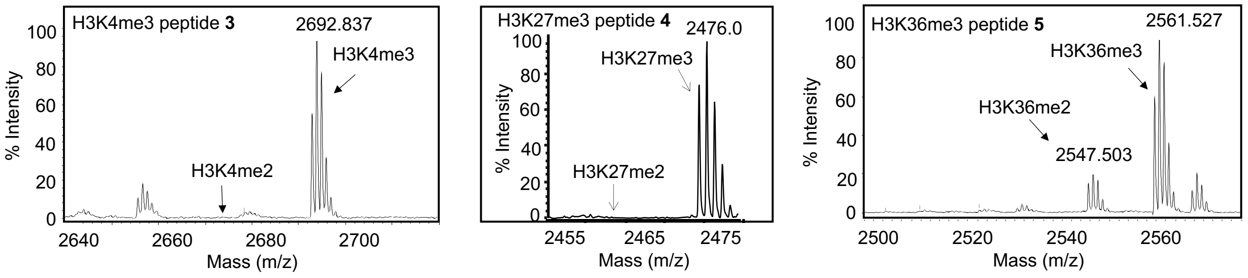

Supplementary Figure S4. MALDI-TOF MS spectra showing activity of KDM4A-I71AzF towards H3K4me3 peptide (**3**), H3K27me3 (**4**) and H3K36me3 (**5**) peptides.

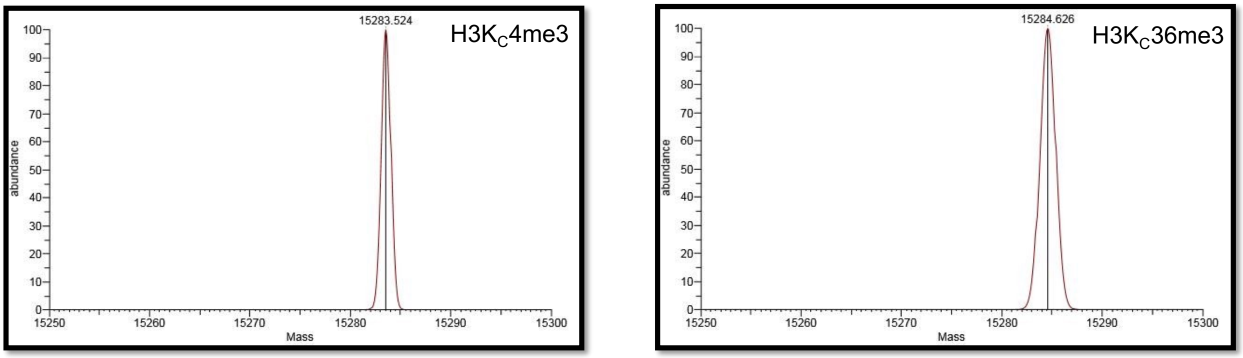

Supplementary Figure S5. Deconvoluted ESI LC-MS spectra of full-length, semi-synthetic histones H3K$_C$4me3 and H3K$_C$36me3.

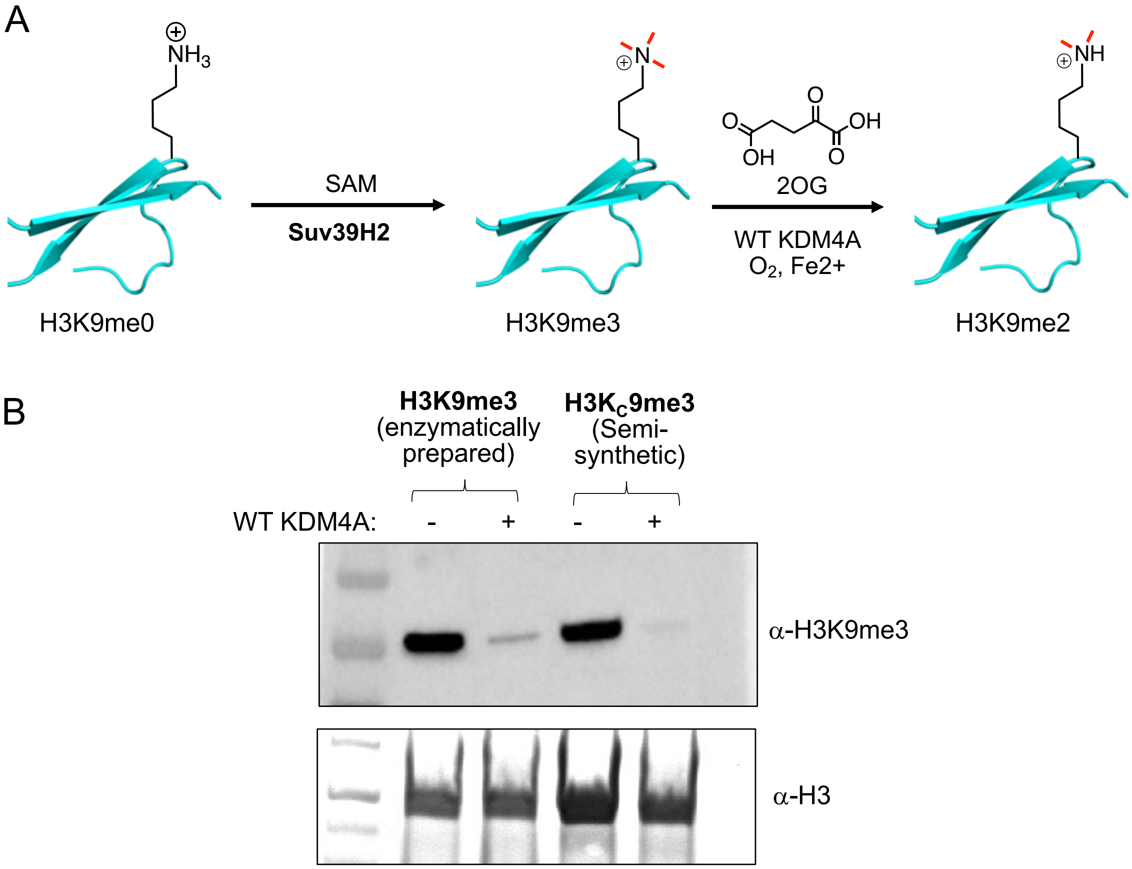

Supplementary Figure S6. (A) Biochemical steps showing tandem activity of SUV39H2 to specifically generate H3K9me3 on full-length H3 using SAM cofactor followed by demethylation to H3K9me2 by wild type KDM4A following a reported method.[17] (B) Western blot analysis of KDM4A-mediated demethylation of H3K9me3 (prepared by SUV39H2) and H3K$_C$9me3 (semi-synthetic) shows KDM4A acts on the both the substrate with equal efficiency.

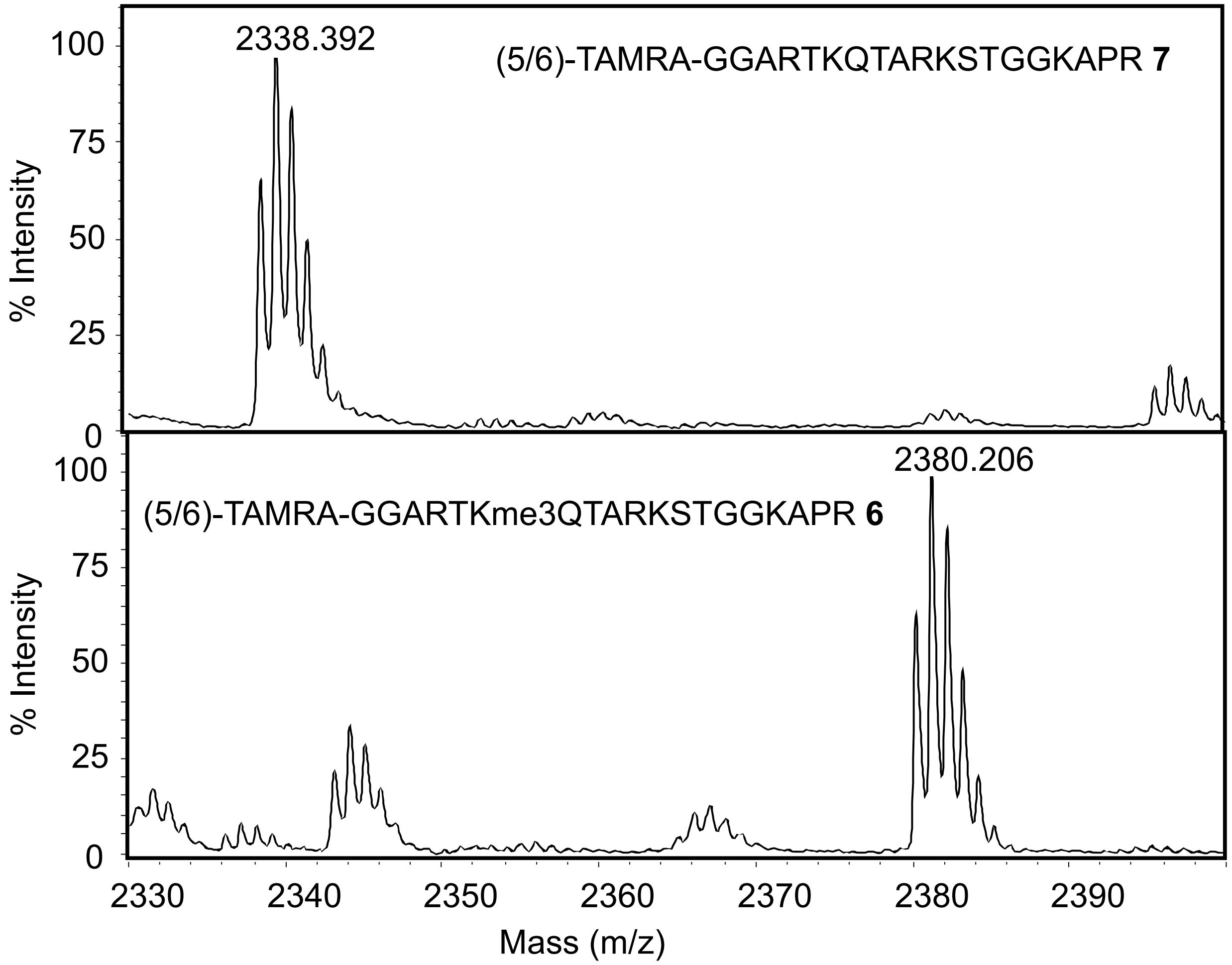

Supplementary Figure S7. MALDI-TOF MS spectra of TAMRA-H3K9me3 **6** and H3K9me0 **7** peptides.

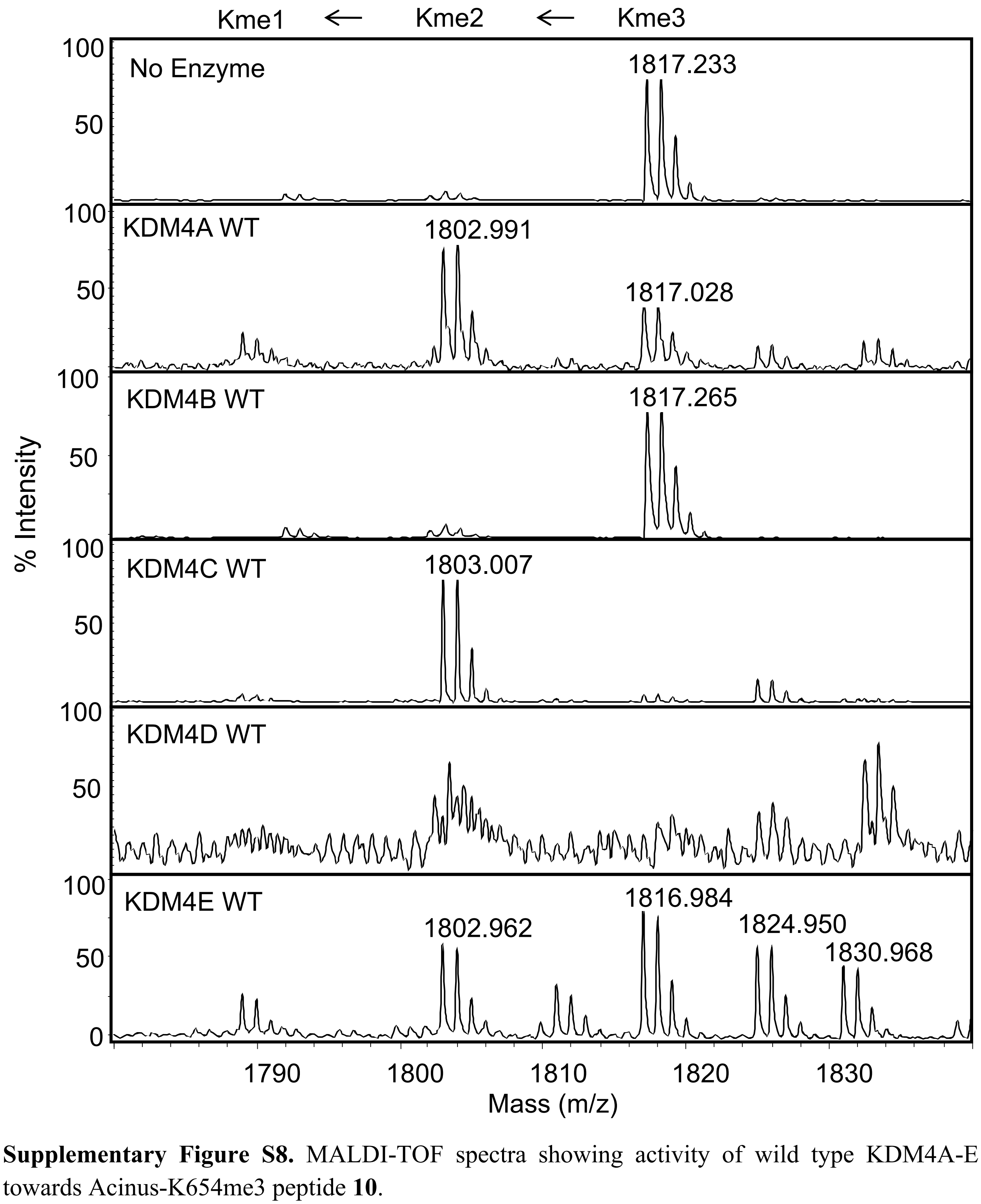

Supplementary Figure S8. MALDI-TOF spectra showing activity of wild type KDM4A-E towards Acinus-K654me3 peptide **10**.

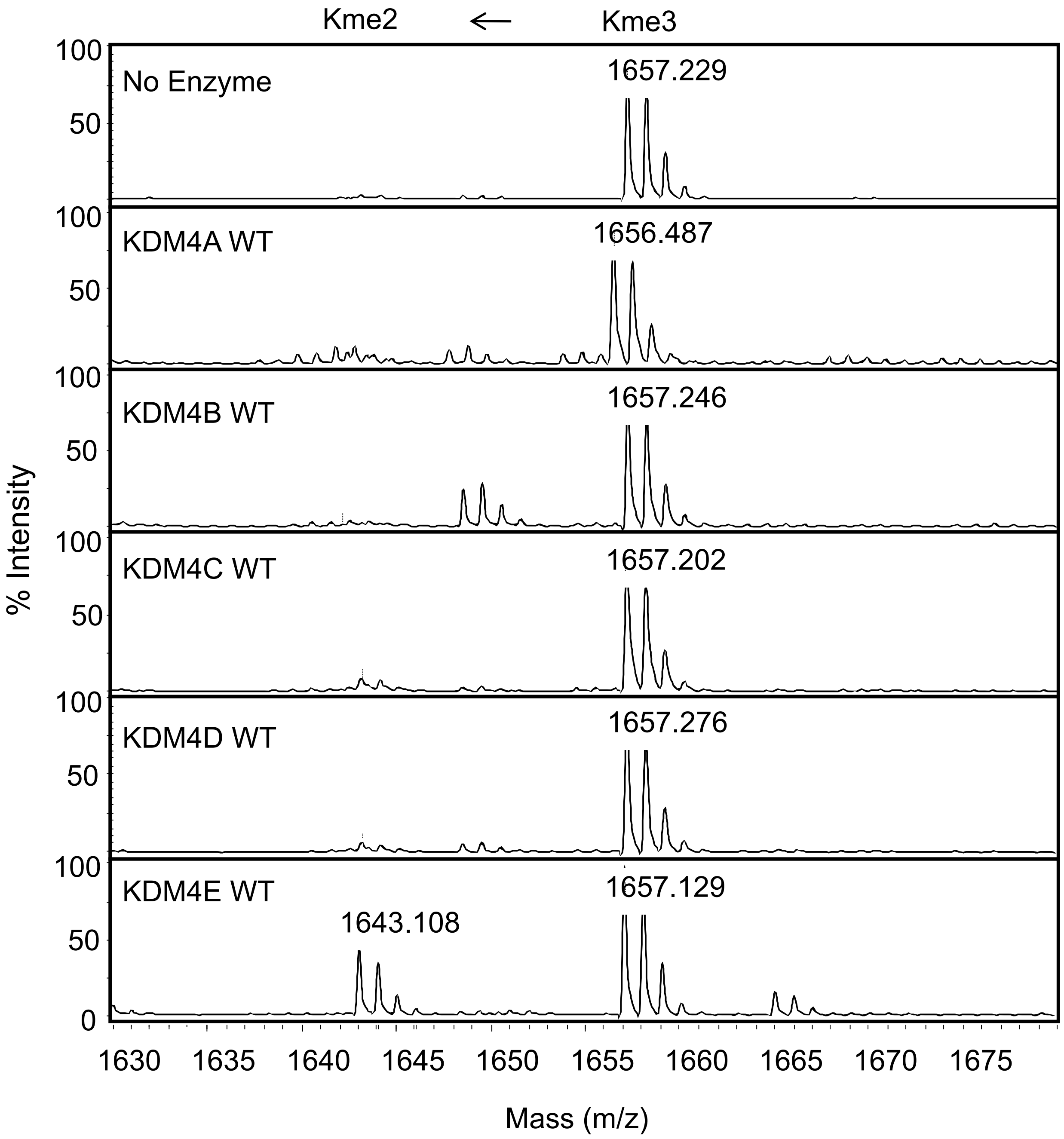

Supplementary Figure S8 Cont'd. MALDI-TOF spectra showing activity of wild type KDM4A-E towards TCPG-K21me3 peptide **15**.

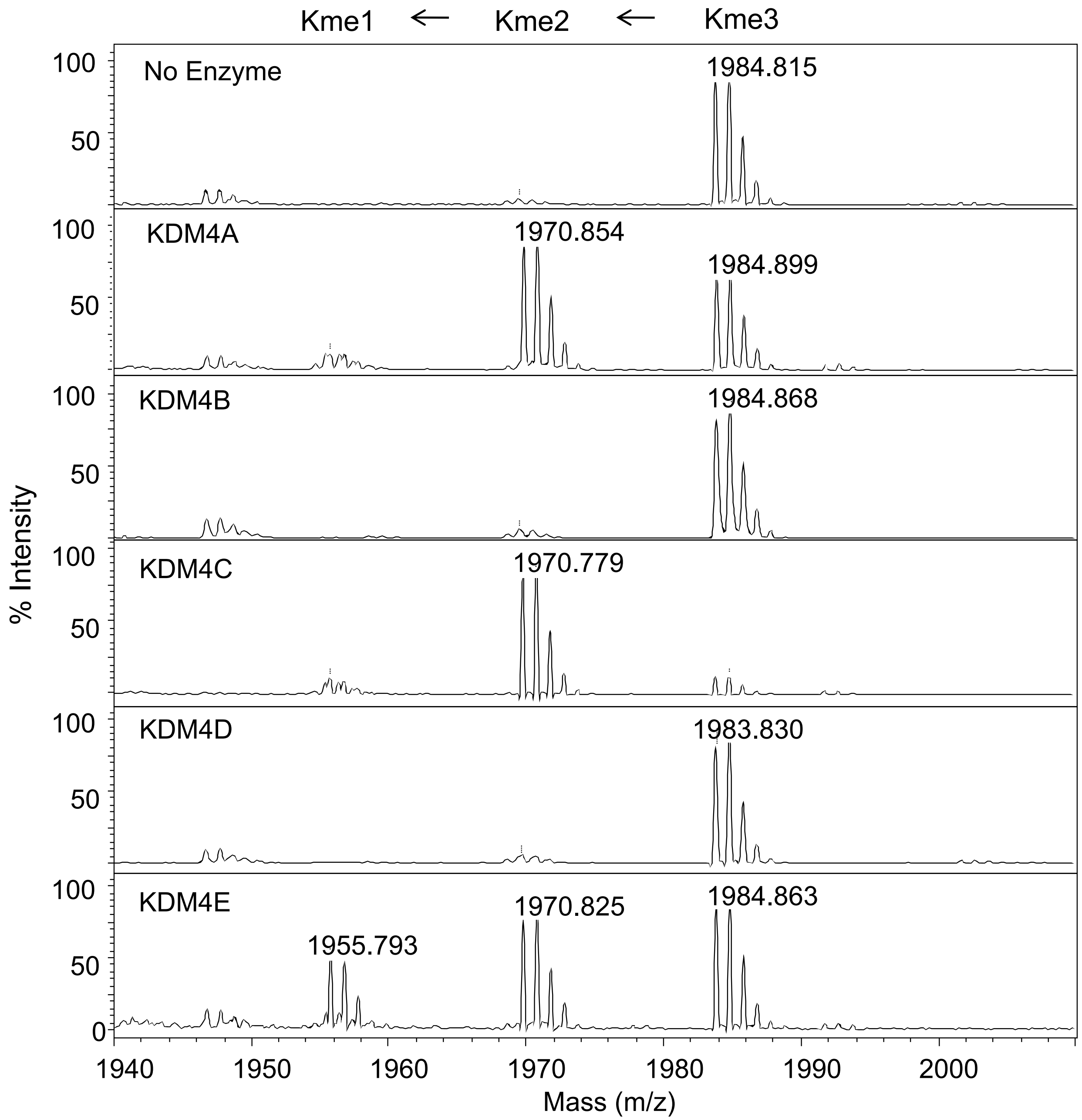

Supplementary Figure S8 Cont'd. MALDI-TOF spectra showing activity of wild type KDM4A-E towards Nup107-K25me3 peptide **16**.

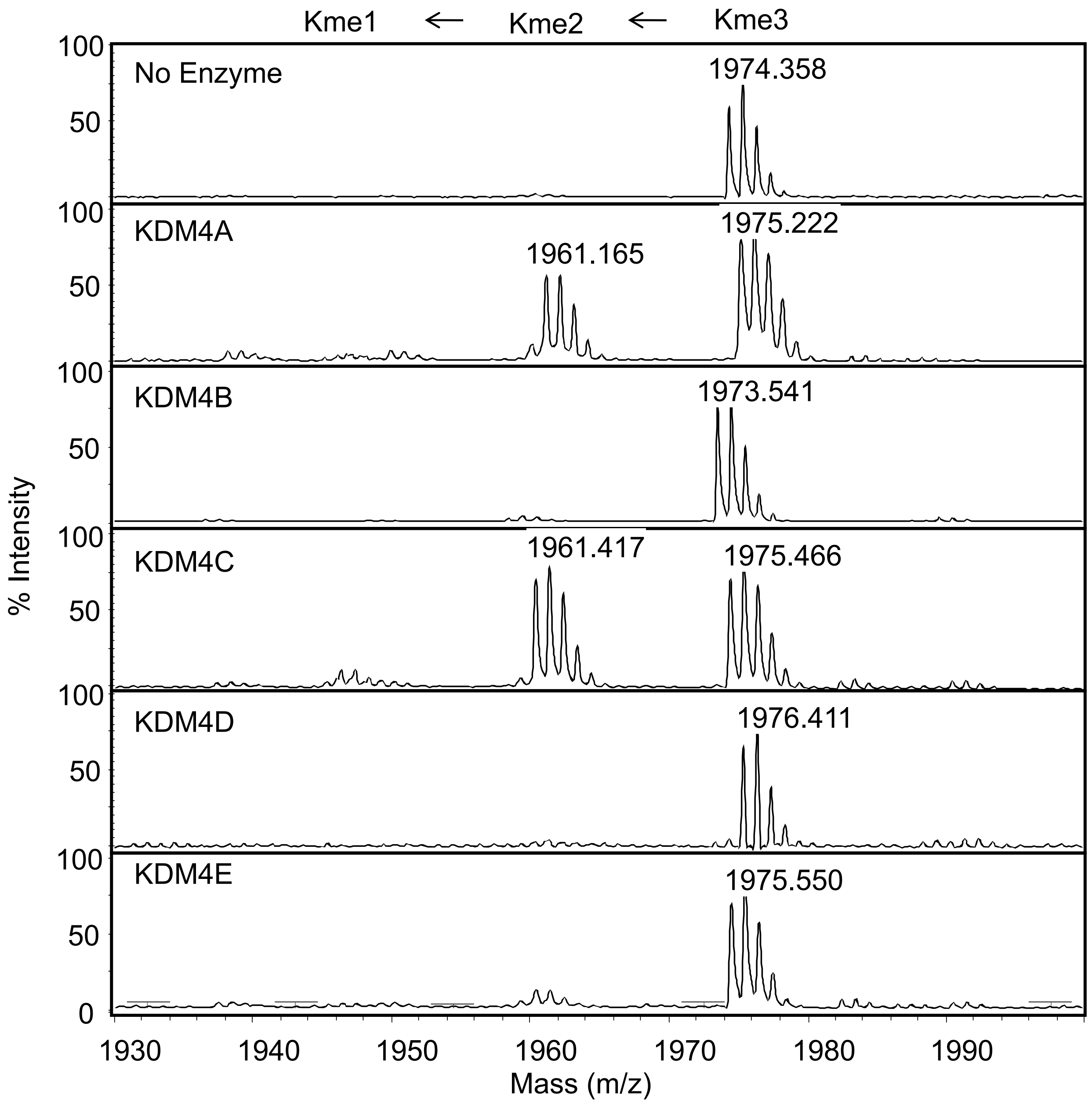

Supplementary Figure S8 Cont'd. MALDI-TOF spectra showing activity of wild type KDM4A-E towards p53-K372me3 peptide **20**.

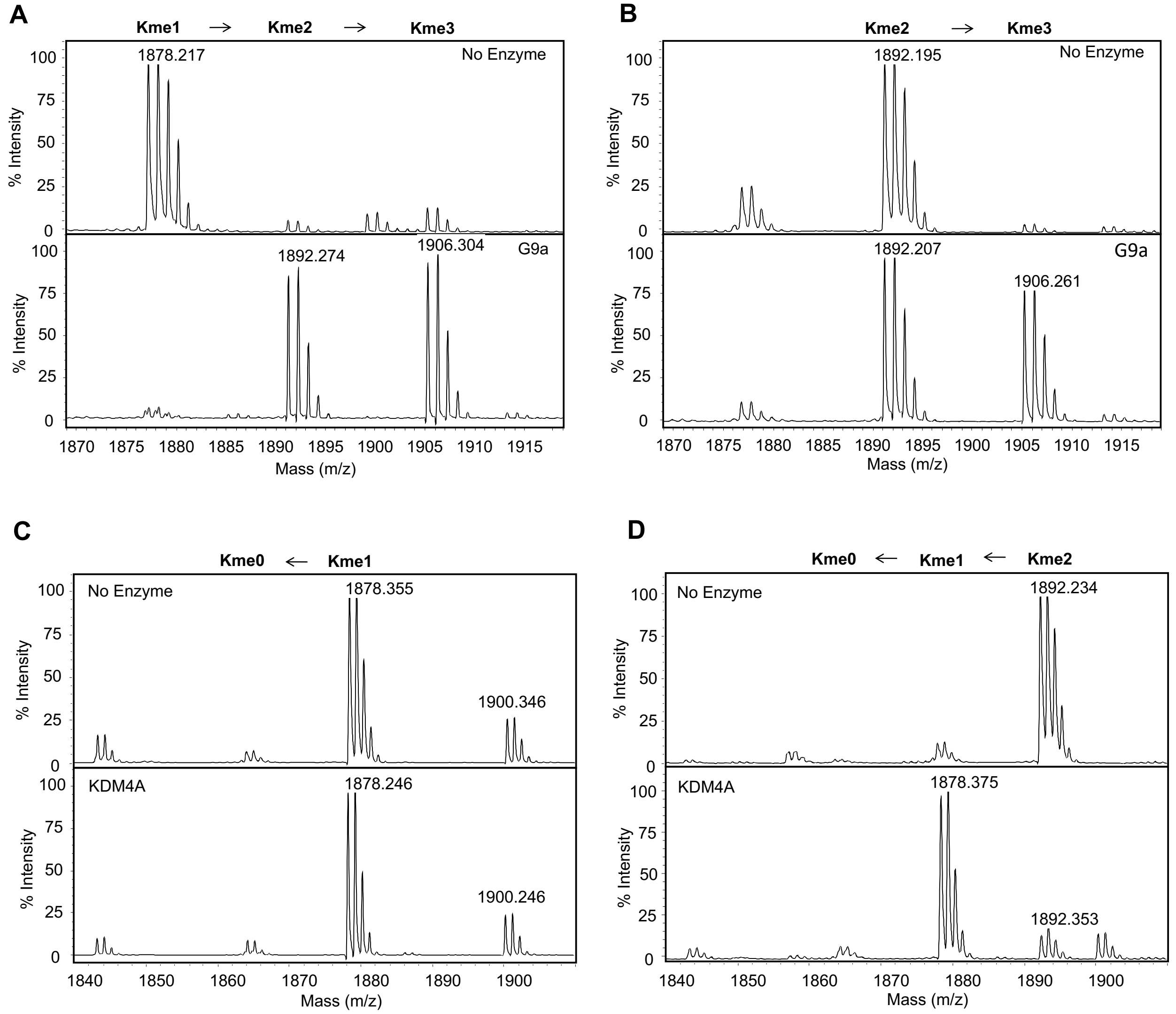

Supplementary Figure S9. MALDI-TOF spectra showing methylation and demethylation of eIF4A3-K374me1 **22** and eIF4A3-K374me2 **23** peptides. (A) eIF4A3-K374me1 peptide **22** is methylated by G9a. (C) eIF4A3-K374me1 peptide **22** is not demethylated by KDM4A. (B) eIF4A3-K374me2 **23** peptide is methylated by G9a. (D) eIF4A3-K374me2 **23** peptide is demethylated only to eIF4A3-K374me1 by KDM4A.

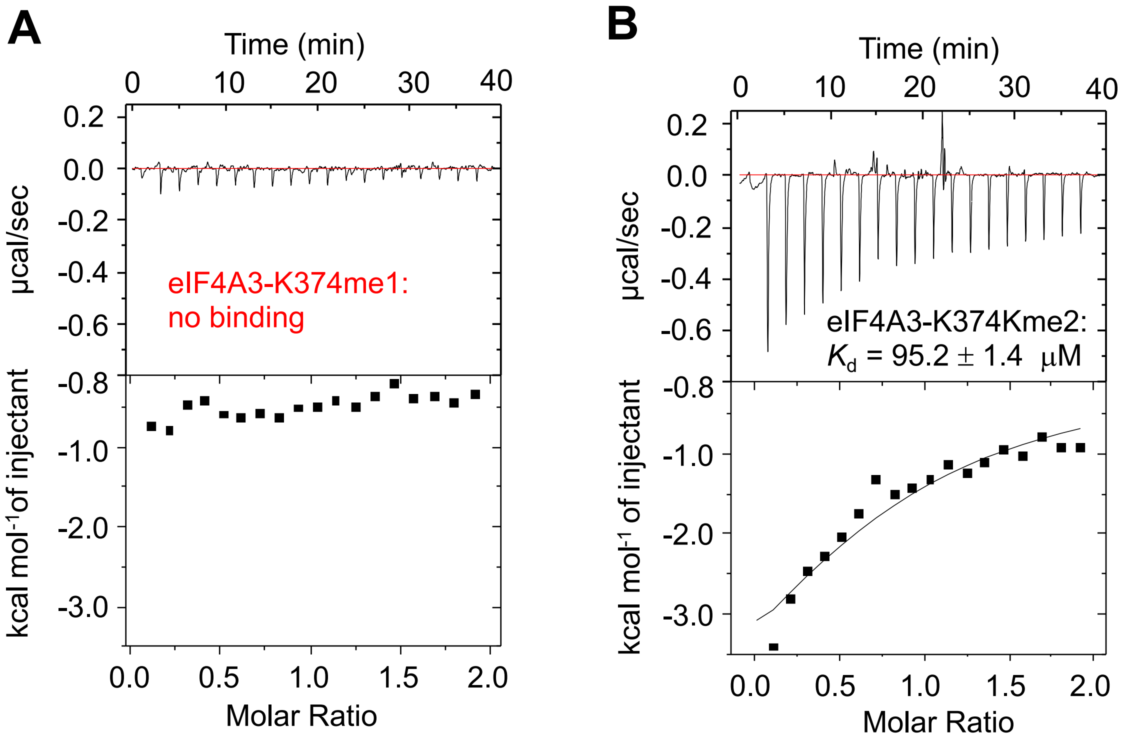

Supplementary Figure S10. Isothermal titration calorimetric (ITC) measurements of dissociation constants of CBX1 chromodomain from eIF4A3 peptides. (A) eIF4A3-K374me1 peptide **22** does not bind to CBX1. (D) eIF4A3-K374me2 peptide **23** binds to CBX1 with K_d of 95.2 ± 1.4 µM.

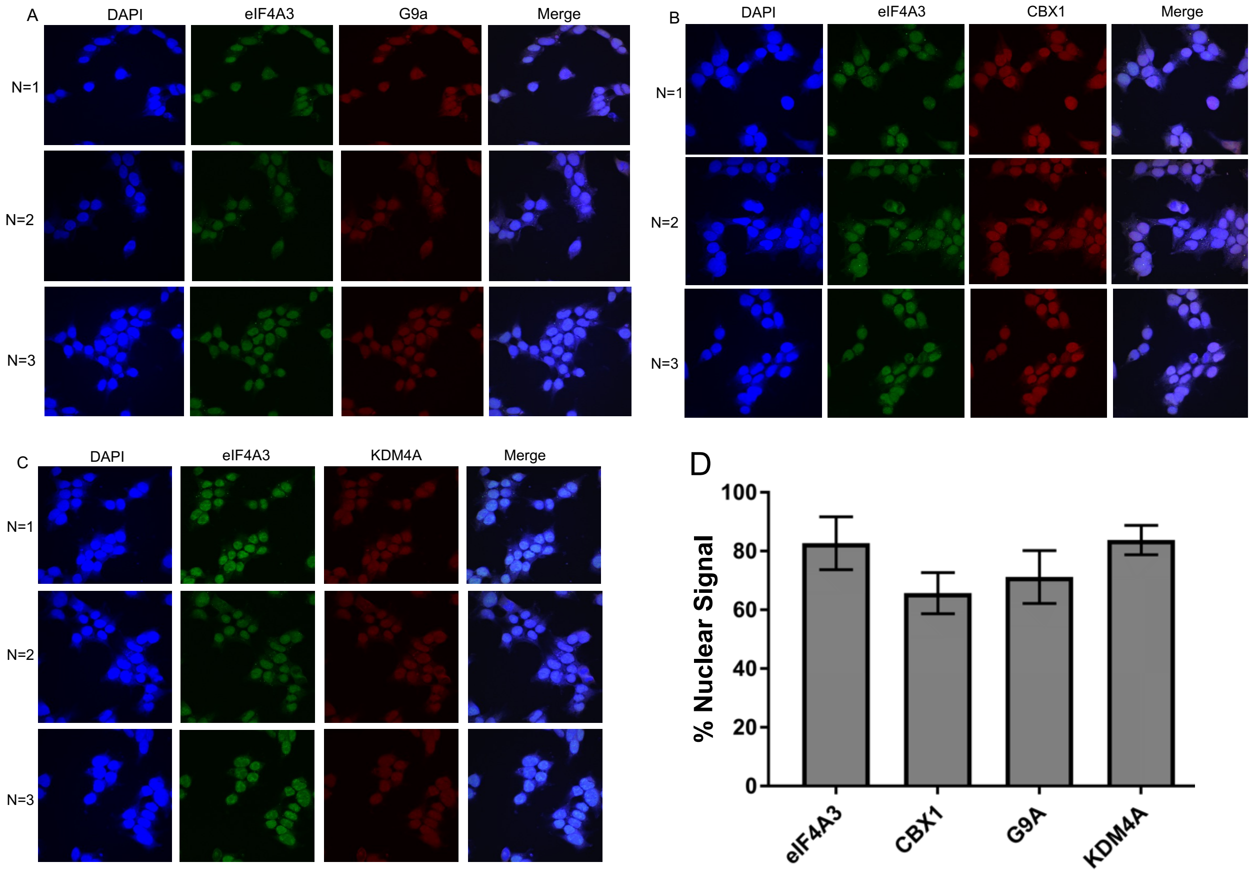

Supplementary Figure S11. Nuclear localization of eIF4A3, CBX1, G9a, and KDM4A in HEK293T cells. The nucleus is identified by DAPI stain in blue and eIF4A3 is depicted in green (A-C). Writer protein G9a (A), reader protein CBX1 (B), and eraser protein KDM4A (C) are all shown in red. The merged panels demonstrate the nuclear compartmentalization of all the proteins. Quantification of the nuclear protein signal was determined and presented as a percentage (D).

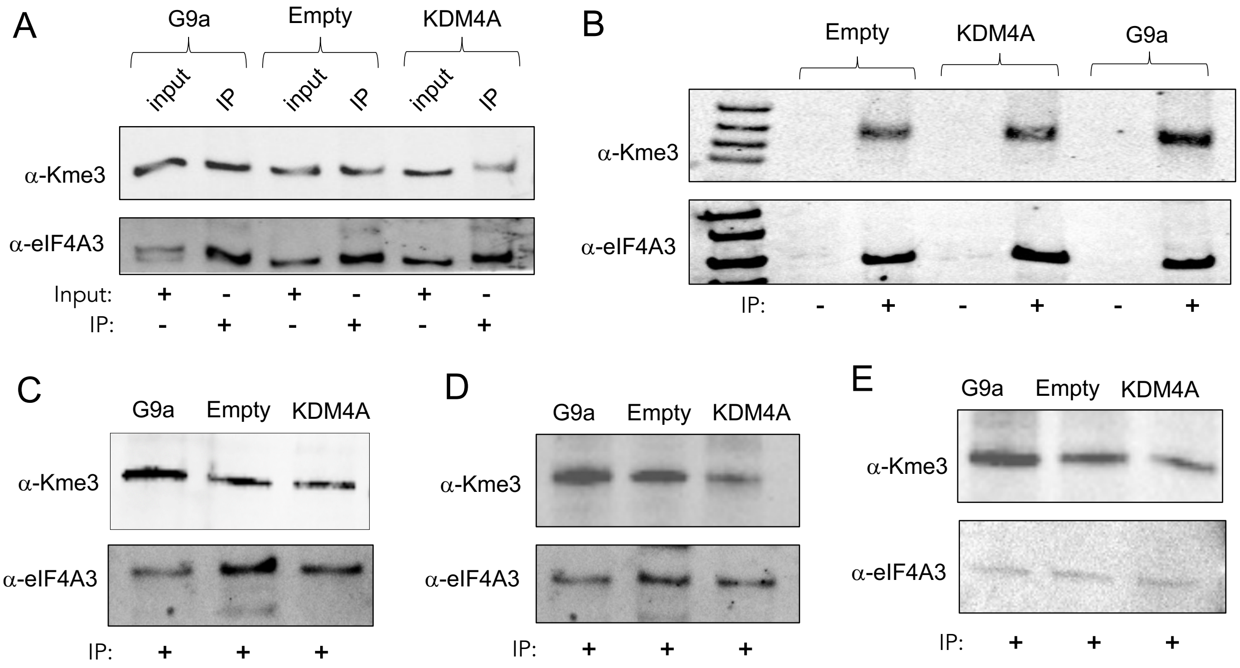

Supplementary Figure S12. Five biological replicates (A - C) demonstrating changes in eIF4A3 trimethylation in HEK293T cells expressing individually control vector, full-length G9a and full-length KDM4A. In each case, cell extracted were immunoprecipitated with eIF4A3 antibody and immunoblotted using the indicated antibodies.

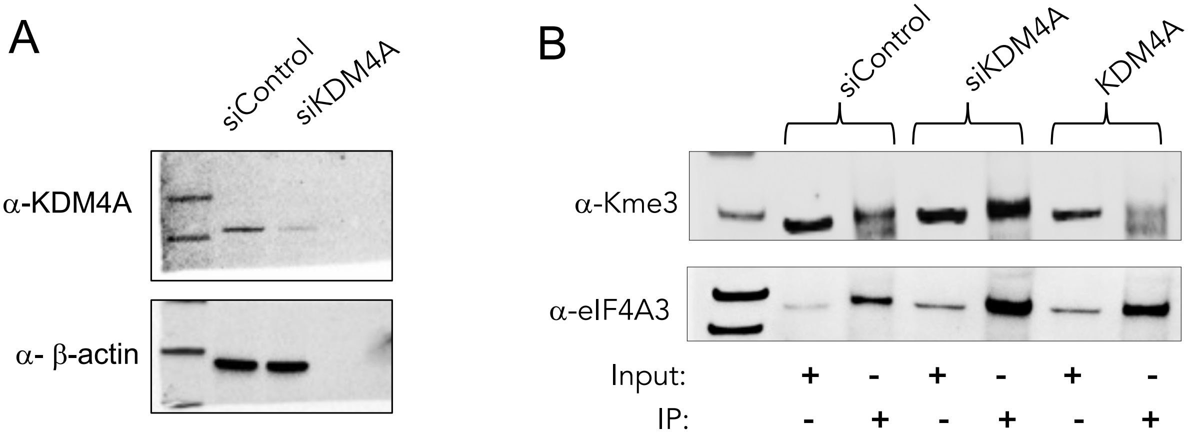

Supplementary Figure S13. (A) Efficient knock down of KDM4A by siRNA as evident from Western blot analysis using KDM4A-specific antibody. (B) A biological replicate demonstrating changes in eIF4A3 trimethylation in HEK293T cells expressing individually control siRNA, siKDM4A and overexpressing wildtype full-length KDM4A. In each case, cell extracted were immunoprecipitated with eIF4A3 antibody and immunoblotted using the indicated antibodies.

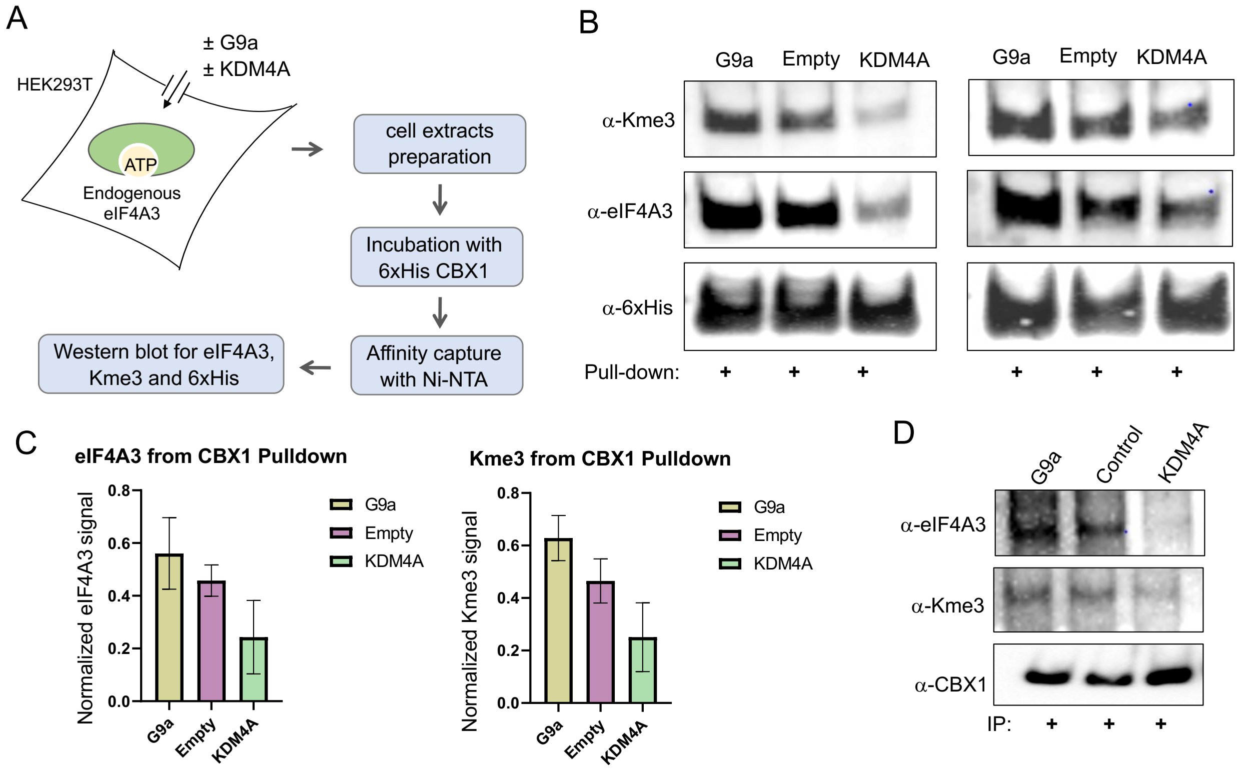

Supplementary Figure S14. (A) Schematic showing pull-down of endogenous eIF4A3 from HEK293T cells expressing control vector, full-length G9a and KDM4A using Ni-NTA coated magnetic beads via bacterially expressed CBX1 Chromodomain carrying 6xHis tag at N-terminus. (B) Western blot of the enriched samples using the indicated antibodies. Left and right panels are two biological replicates. (C) Bar diagrams representing quantification of changes in eIF4A3 enrichment and its methylation level. (D) Biological replicate of eIF4A3 enrichment by endogenous CBX1 from HEK293T cells expressing control vector, full-length G9a and KDM4A.

Protein Name	Amino Acid Molecular Weight	AzF Molecular weight	Modification	Molecular Weight difference from WT	Calculated Molecular Weight	Observed Molecular weight	Difference (WT-AzF protein) - based on LC-HRMS
KDM4A WT	-	-	-	-	44236.92	44263.914	
KDM4A I71AzF	Ile-131.17	206.2	I71 to pAzF	75.03	44311.95	44338.844	74.93
KDM4A V171AzF	Val-117.15	206.2	V171 to pAzF	89.05	44325.97	44352.531	88.617
KDM4A Y175AzF	Tyr-181.19	206.2	Y175 to pAzF	25.01	44261.93	44288.074	24.16
KDM4A Y177AzF	Tyr-181.19	206.2	Y177 to pAzF	25.01	44261.93	44289.16	25.246
KDM4A D191AzF	Asp-133.10	206.2	D191 to pAzF	73.1	44310.02	44336.95	73.036
KDM4A S288AzF	Ser-105.09	206.2	S288 to pAzF	101.11	44338.03	44364.668	100.754
KDM4A N290AzF	Asn-132.11	206.2	N290 to pAzF	74.09	44311.01	44337.652	73.738
KDM4A V313AzF	Val-117.15	206.2	V313 to pAzF	89.09	44326.01	44381.4	117.486
KDM4C WT	-	206.2	-	-	43509.3	43405.32	
KDM4C I73AzF	Ile-131.17	206.2	I73 to pAzF	75.03	43584.33	43480.46	75.14
KDM4D WT	-	206.2	-	-	40790.06	41104.895	
KDM4D L75AzF	Leu-131.17	206.2	L75 to pAzF	75.03	40865.09	41179.02	74.125
KDM4E WT	-	206.2	-	-	41120.84	41146.332	
KDM4E L72AzF	Leu-131.17	206.2	L72 to pAzF	75.03	41195.87	41220.77	74.438

Supplementary Table S1. Calculated and experimental molecular weights of wild type KDM4 proteins and their AzF mutants. The additional 28Da observed for KDM4A-V313AzF is likely due to N-terminus formylation.

Peptide Name	Compound #	Peptide Sequence
H3K9me3	**2**	ARTKQTARKme3STGGKAPRKQLA
H3K4me3	**3**	ARTKme3QTARKSTGGKAPRKQLK
H3K27me3	**4**	APRKQLATKAARKme3SAPATGGVK
H3K36me3	**5**	KSAPSTGGVKme3KPHRYKPGTGK
TAMRA-H3K9me3	**6**	(5/6)-TAMRA-GGARTKQTARKme3STGGKAPR
TAMRA-H3K9me0	**7**	(5/6)-TAMRA-GGARTKQTARKSTGGKAPR
AIFM1-K590me3	**8**	FNRMPIARKme3IIKDGEQH
ACINUS-K113me3	**9**	ALMLENLQKme3HSTPHAAF
ACINUS-K654me3	**10**	RSASSNSRKme3SLSPGVSR
hnRNPQ-K125me3	**11**	KGPDEAKIKme3ALLERTGY
SF3B1-K290me3	**12**	GGATSSARKme3NRWDEPTK
eIF4A3-K374me3	**13**	GRSGRYGRKme3GVAINFVK
SYDC-K9me3	**14**	MPSASASRKme3SQEKPREI
TCPG-K21me3	**15**	NTKRESGRKme3VQSGNINA
Nup107-K25me3	**16**	AEVTRTARKme3QSAQKRVL
NUP107-K305me3	**17**	WENTLHTLKme3QRQLTSYV
P53-K370me3	**18**	SRAHSSHLKme3SKKGQSTS
P53-K372me3	**19**	AHSSHLKSKme3KGQSTSRH
P53-K373me3	**20**	HSSHLKSKKme3GQSTSRHK
eIF4A3-K374me0	**21**	GRSGRYGRKGVAINFVK
eIF4A3-K374me1	**22**	GRSGRYGRKme1GVAINFVK
eIF4A3 K374me2	**23**	GRSGRYGRKme2GVAINFVK

Supplementary Table S2. List of the synthesized peptides. Each peptide carries a C-terminus amide moiety. Peptide numbers are based on their first appearance in the manuscript.

Supplementary Table S3 contains analyzed proteomic data and provided in Excel file separately.

Gene	Expression Vector	Antibiotic Resistance	Affinity Tag
KDM4A	pNIC28-Bsa4	Kanamycin	N-6xHis
KDM4A	pNIC28-Bsa4	Kanamycin	N-Strep (II)
KDM4B	pST4	Ampicillin	N-6xHis
KDM4C	pST4	Ampicillin	N-Strep (II)
KDM4D	pST4	Ampicillin	N-Strep (II)
KDM4E	pNIC28-Bsa4	Kanamycin	N-His-TEV
CBX1_LEEEEE	pET28-MHL	Kanamycin	N-6xHis
G9a	pET28a	Kanamycin	N-6xHis
SUV-3,9-H2	pET28a	Kanamycin	N-6xHis
pEVOL-pAzF	P15A	Chloramphenicol	N/A
hKDM4A	pCMV	Kanamycin	N-HA
hG9a	pcDNA3.1	Ampicillin	N-HA
CaMKMT	pET28-MHL	Kanamycin	N-6xHis
DOT1L	pET28-MHL	Kanamycin	N-6xHis-TEV
METTL20	pET28a	Kanamycin	N-6xHis
METL21A	pET28-MHL	Kanamycin	N-6xHis
METL21C	pET28-MHL	Kanamycin	N-6xHis

Supplementary Table S4. List of the genes used in the current study. The expression vector, antibiotic resistance and the affinity tag present for protein purification are provided.

Primer Name	Sequence
KDM4A I71TAG	5'- GGTCATTCCTGCCCCCTAGCAACAGCTGGTGACG -3'
KDM4A V171TAG	5'- GGATCACCATTGAGGGTTAGAACACCCCATACCTG -3'
KDM4A Y175TAG	5'- GGTGTGAACACCCCATAGCTGTACTTTGGCATG -3'
KDM4A Y177TAG	5'- GAACACCCCATACCTGTAGTTTGGCATGTGGAAG -3'
KDM4A D191TAG	5'- GCTTGGCACACTGAATAGATGGACCTCTACAGC -3'
KDM4A S288TAG	5'- GGTTTTAACTGTGCGGAGTAGACCAATTTTGCTACCCG -3'
KDM4A N290TAG	5'- CTGTGCGGAGTCTACCTAGTTTGCTACCCGTCGG -3'
KDM4A V313TAG	5'- GCTCCTGTAGAAAGGACATGTAGAAGATCTCCATGG -3'
KDM4B I72TAG	5'- GATCCCGGCGCCCTAGCAGCAGGTG -3'
KDM4C I73TAG	5’-ATTTGCTCATTCCAGCACCATAGCAGCAGATGGTCACA-3’
KDM4D L75AzF	5’-ATCTTAATAGCCACTCCCTAGCAGCAGGTGGCCTCTGGG-3’
KDM4E L72AzF	5’CATCTTAATAGCCACTCCCTAGCAGCAGGTGACCTCTGGGC-3’
H3 K4C	5'-GCGCGTACTTGCCAGACGGCTCG-3'
H3 K9C	5’-GCAGACGGCTCGGTGCTCCACCGGCGG-3’
H3 K36C	5'-CTACCGGCGGCGTGTGCAAGCCTCACCGTTAC-3'

Supplementary Table S5. List of primers designed for site-directed mutagenesis. Reverse primers used are the reverse-complement to the given forward primers.

Peptide	T (K)	ΔS cal/mol/degree	TΔS (kcal/mol)	ΔH (kcal/mol)	ΔG (kcal/mol)
eIF4A3_K374me0 **21**	288.15	0	0	0	0
eIF4A3_K374me1 **22**	288.15	0	0	0	0
eIF4A3_K374me2 **23**	288.15	-3.84	-1.106	-6.576	-5.470
eIF4A3_K374me3 **13**	288.15	8.58	2.472	-3.758	-6.230

Supplementary Table S6. Thermodynamic parameters of CBX1 binding to eIF4A3-K374me0/1/2/3 peptides as evaluated by isothermal titration calorimetry.

26. References

(1) Sudhamalla, B.; Dey, D.; Breski, M.; Nguyen, T.; Islam, K., Site-specific azide-acetyllysine photochemistry on epigenetic readers for interactome profiling. *Chemical science (Royal Society of Chemistry : 2010)* **2017,** *8* (6), 4250-4256.

(2) Arora, S.; Horne, W. S.; Islam, K., Engineering Methyllysine Writers and Readers for Allele-Specific Regulation of Protein-Protein Interactions. *Journal of the American Chemical Society* **2019,** *141* (39), 15466-15470.

(3) Huang, Z.-P.; Du, J.-T.; Zhao, Y.-F.; Li, Y.-M., Synthesis of Site-Specifically Dimethylated and Trimethylated Peptides Derived from Histone H3 N-Terminal Tail. *International Journal of Peptide Research and Therapeutics* **2006,** *12* (2), 187-193.

(4) Breski, M.; Dey, D.; Obringer, S.; Sudhamalla, B.; Islam, K., Engineering Biological C–H Functionalization Leads to Allele-Specific Regulation of Histone Demethylases. *Journal of the American Chemical Society* **2016,** *138* (41), 13505-13508.

(5) Krishnan, S.; Trievel, R. C., Structural and functional analysis of JMJD2D reveals molecular basis for site-specific demethylation among JMJD2 demethylases. *Structure (London, England : 1993)* **2013,** *21* (1), 98-108.

(6) Krishnan, S.; Collazo, E.; Ortiz-Tello, P. A.; Trievel, R. C., Purification and assay protocols for obtaining highly active Jumonji C demethylases. *Analytical biochemistry* **2012,** *420* (1), 48-53.

(7) Pack, L. R.; Yamamoto, K. R.; Fujimori, D. G., Opposing Chromatin Signals Direct and Regulate the Activity of Lysine Demethylase 4C (KDM4C). *The Journal of biological chemistry* **2016,** *291* (12), 6060-70.

(8) Scott, V.; Dey, D.; Kuwik, J.; Hinkelman, K.; Waldman, M.; Islam, K., Allele-Specific Chemical Rescue of Histone Demethylases Using Abiotic Cofactors. *ACS chemical biology* **2021**.

(9) Chin, J. W.; Martin, A. B.; King, D. S.; Wang, L.; Schultz, P. G., Addition of a photocrosslinking amino acid to the genetic code of Escherichiacoli. *Proceedings of the National Academy of Sciences of the United States of America* **2002,** *99* (17), 11020-4.

(10) Simon, M. D.; Chu, F.; Racki, L. R.; de la Cruz, C. C.; Burlingame, A. L.; Panning, B.; Narlikar, G. J.; Shokat, K. M., The site-specific installation of methyl-lysine analogs into recombinant histones. *Cell* **2007,** *128* (5), 1003-12.

(11) Simon, M. D., Installation of site-specific methylation into histones using methyl lysine analogs. *Current protocols in molecular biology / edited by Frederick M. Ausubel ... [et al.]* **2010,** *Chapter 21*, Unit 21.18.1-10.

(12) Hopkinson, R. J.; Tumber, A.; Yapp, C.; Chowdhury, R.; Aik, W.; Che, K. H.; Li, X. S.; Kristensen, J. B.; King, O. N.; Chan, M. C.; Yeoh, K. K.; Choi, H.; Walport, L. J.; Thinnes, C. C.; Bush, J. T.; Lejeune, C.; Rydzik, A. M.; Rose, N. R.; Bagg, E. A.; McDonough, M. A.; Krojer, T.; Yue, W. W.; Ng, S. S.; Olsen, L.; Brennan, P. E.; Oppermann, U.; Muller-Knapp, S.; Klose, R. J.; Ratcliffe, P. J.; Schofield, C. J.; Kawamura, A., 5-Carboxy-8-hydroxyquinoline is a Broad Spectrum 2-Oxoglutarate Oxygenase Inhibitor which Causes Iron Translocation. *Chemical science (Royal Society of Chemistry : 2010)* **2013,** *4* (8), 3110-3117.

(13) King, O. N.; Li, X. S.; Sakurai, M.; Kawamura, A.; Rose, N. R.; Ng, S. S.; Quinn, A. M.; Rai, G.; Mott, B. T.; Beswick, P.; Klose, R. J.; Oppermann, U.; Jadhav, A.; Heightman, T. D.; Maloney, D. J.; Schofield, C. J.; Simeonov, A., Quantitative high-throughput screening identifies 8-hydroxyquinolines as cell-active histone demethylase inhibitors. *PloS one* **2010,** *5* (11), e15535.

(14) Shevchenko, A.; Tomas, H.; Havlis, J.; Olsen, J. V.; Mann, M., In-gel digestion for mass spectrometric characterization of proteins and proteomes. *Nature protocols* **2006,** *1* (6), 2856-60.
(15) Van Rechem, C.; Black, J. C.; Boukhali, M.; Aryee, M. J.; Graslund, S.; Haas, W.; Benes, C. H.; Whetstine, J. R., Lysine demethylase KDM4A associates with translation machinery and regulates protein synthesis. *Cancer discovery* **2015,** *5* (3), 255-63.
(16) Johansson, C.; Tumber, A.; Che, K.; Cain, P.; Nowak, R.; Gileadi, C.; Oppermann, U., The roles of Jumonji-type oxygenases in human disease. *Epigenomics* **2014,** *6* (1), 89-120.
(17) Scott, V.; Dey, D.; Kuwik, J.; Hinkelman, K.; Waldman, M.; Islam, K., Allele-Specific Chemical Rescue of Histone Demethylases Using Abiotic Cofactors. *ACS chemical biology* **2022,** *17* (12), 3321-3330.
